# 24-hour *ex vivo* perfusion of vascularized composite allografts in a large animal total limb model

**DOI:** 10.3389/frtra.2026.1718484

**Published:** 2026-02-12

**Authors:** Çağdaş Duru, Alina Stoian, Felor Biniazan, Florian Le Billan, Golnaz Karoubi, Shaf Keshavjee, Siba Haykal

**Affiliations:** 1 Latner Thoracic Research Laboratories, Division of Thoracic Surgery, Toronto General Hospital, University Health Network, University of Toronto, Toronto, ON, Canada; 2 Division of Plastic and Reconstructive Surgery, Department of Surgery, Yale University, New Haven, CT, United States

**Keywords:** *ex vivo* limb perfusion, *ex vivo* perfusion, hand transplantation, ischemia - reperfusion, vascularized composite allograft (VCA)

## Abstract

**Background:**

Static cold storage (SCS) at 4 °C remains the standard for preserving vascularized composite allotransplants (VCA) but limits viability to approximately 6 h. *Ex-vivo* perfusion offers a promising alternative. This study presents a 24-h sub-normothermic perfusion protocol in a swine hindlimb model using autologous red blood cells (RBCs).

**Methods:**

Limbs perfused for 24 h were compared with cold-stored limbs. The perfusate contained LPD, 2.5 g/dlBSA, heparin, methylprednisolone, dextrose, insulin, L-alanyl L-glutamine, sodium bicarbonate, and washed RBCs (hematocrit 10%–15%). Perfusion was maintained at 60–65 mmHg and 28 °C–32 °C. Perfusate was monitored hourly. Biopsies (thigh and distal foot) were collected every 6 h for histology and ATP. Limbs were weighed at baseline and endpoint.

**Results:**

Perfusion preserved stable ATP in proximal muscle (0.416–0.367 nmol/μl) and distal muscle (0.315–0.267 nmol/μl). In contrast, SCS showed significant ATP depletion in proximal muscle (0.502–0.15 nmol/μl, *p* = 0.0086) and distal muscle (0.335–0.078 nmol/μl, *p* = 0.0216). Injury scores corroborated these findings. In proximal muscle, scores remained stable with perfusion (3.03–3.26) but increased with SCS (2.4 increasing to 3.73, *p* = 0.0079). In the distal muscle, scores rose in both groups (perfusion: 2.90 increasing to 4.63; SCS: 2.56 increasing to 4.0), with significance only in the control group (*p* = 0.0291). Limb weight was unchanged (–0.53% perfusion vs. −0.62% SCS).

**Conclusion:**

Twenty-four-hour swine hindlimb perfusion preserved ATP, morphology, and function. Perfusion prevented ATP depletion and mitigated muscle damage compared with SCS, supporting its potential to extend VCA preservation. Transplant studies are warranted.

## 1 Introduction

Upper extremity loss, defined as amputation above the wrist, profoundly impacts patients by limiting daily function and causing significant psychological challenges (1). The first successful hand transplant in 1998 marked a turning point, proving the potential of vascularized composite allotransplantation (VCA) to restore form and function. Since then, over 148 hand and upper extremity transplants have been reported worldwide, highlighting growing clinical interest in VCA as a reconstructive option (2).

Currently, the standard method for preserving large extremity segments, whether for transplantation or traumatic amputations, is static cold storage (SCS) at 4 °C (3). This slows metabolism but does not entirely stop it (4); leading to energy depletion, ion imbalance, cellular swelling, and mitochondrial dysfunction, which worsen tissue damage and immune response upon reperfusion (5–7). Muscle tissue, abundant in extremities, is particularly ischemia-sensitive (8). Muscle damage occurs after six hours of cold storage, and ischemia beyond 12 h generally precludes major limb salvage (9). Similarly, in VCA, ischemia over 4–6 h reduces functional recovery, limiting geographic feasibility and clinical application (9, 10).

To overcome the limitations of static cold storage, *ex vivo* machine perfusion has become an established preservation technique in lung, kidney, liver, and heart transplantation (11–15). It expands the donor pool by enabling better organ assessment, prolonging tolerable ischemia time, and reducing ischemia-reperfusion injury (16, 17).

Despite its success in solid organ transplantation, *ex vivo* perfusion has yet to achieve clinical translation in VCA. With growing interest in reconstructive transplantation, experimental strategies have been tested in small and large animal models under hypothermic (0 °C–13 °C), sub-normothermic (13 °C–34 °C), and normothermic (34 °C–38 °C) conditions, each showing advantages over static cold storage (18–22). A key challenge for extended perfusion is weight increase (23). A weight increase of 20% of the initial weight has been associated with compartment pressures above 30 mmHg (24), leading to iatrogenic compartment syndrome. Such pressure elevations can lead to microvascular compromise after reperfusion, thereby impairing graft outcomes through muscle injury. Consistent with this, additional edema after reperfusion has been reported in experimental studies that include a reperfusion phase (21, 25, 26) as well as in clinical transplants (27). These findings underscore weight gain as a critical barrier to prolonged limb preservation, indicating that any clinically translatable machine perfusion strategy must minimize weight gain.

Therefore, the objective of this study was to develop an *ex vivo* machine perfusion protocol capable of preserving a whole swine hindlimb while minimizing weight gain within intact fascial compartments. To achieve this, we employed a novel, modified low-potassium dextran–based perfusate supplemented with red blood cells and operated under low-flow conditions. This model represents a substantially larger and anatomically constrained tissue mass than previously reported systems, thereby more closely approximating clinically relevant extremity preservation. We compared this approach with static cold storage to establish a clinically translatable platform with the potential to extend ischemia tolerance and improve outcomes in extremity transplantation and limb salvage.

## 2 Methods

### 2.1 Animal use

**Figure 1:**
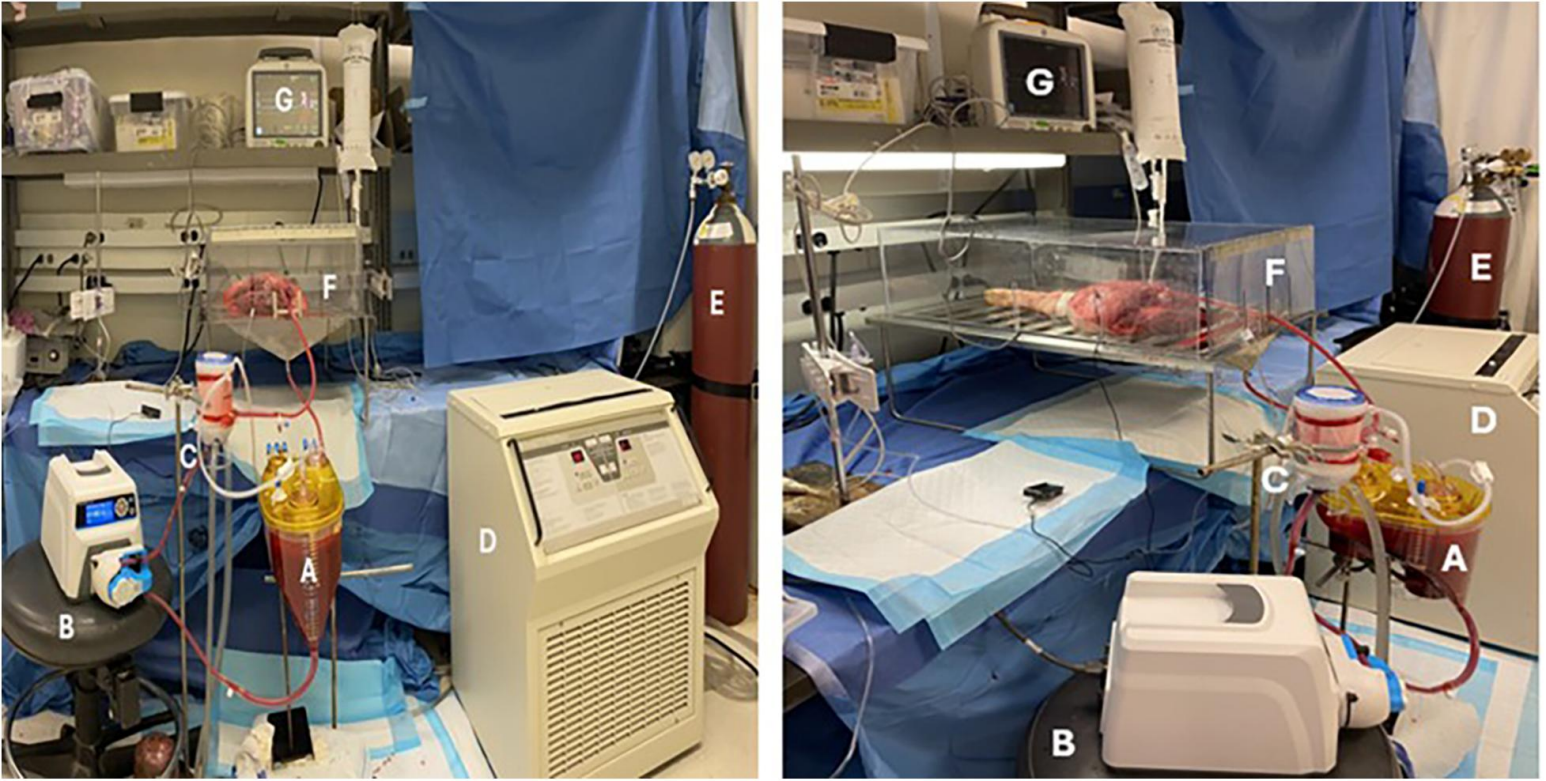
The *ex vivo* perfusion set-up **(A)** reservoir (eurosets RemoweLL2 oxygenator/reservoir, nipro Canada), **(B)** peristaltic pump (masterflex® L/S® digital drive with easy-load® 3 pump head, 115/230 VAC), components were connected using sterile silicone tubing (masterflex® L/S® precision pump tubing, platinum-cured silicone, 3/8″ (size 17, for pump and arterial line) and 1/4″ (size 18, connection from the reservoir outlet). Length of the tubing was standardized and for each experiment new tubing set was precisely cut and sterilized. **(C)** Oxygenator (Eurosets RemoweLL2 Oxygenator/Reservoir, Nipro Canada), **(D)** Heater-cooler (CSZ Hemotherm 400MR, USA), **(E)** Carbogen gas tank, **(F)** Custom limb receptacle, **(G)** Monitor (General Electric Transport Pro, USA).

The animal surgeries were performed in the Latner Thoracic Laboratories in the Toronto General Hospital Research Institute. All experiments were approved by the Institutional Animal Care and Use Committee (IACUC) of University Health Network and the Toronto General Hospital Research Institute under animal use protocol number AUP6474.11. 10 male Yorkshire pigs weighing between 33 and 43 kg were used for the study. Five 24-h perfused limbs were compared to five static cold-stored (SCS) controls that were preserved for 12 h. In preliminary studies, 24 h of SCS resulted in severe graft injury and was not feasible to study [Supplementary File; Figure 1 shows proximal and distal muscle H&E sections after 24-h cold static preservation with Low Potassium Dextran (LPD) solution].

### 2.2 Limb procurement and blood collection

Animals were sedated with ketamine (20 mg/kg IM), atropine (0.04 mg/kg IM), and midazolam (0.3 mg/kg IM). Anesthesia was induced with inhaled isoflurane (mask, 22–44 ml/kg/min, 5%). After intubation, pigs were positioned supine and heparinized intravenously (10,000 IU). The field was prepped with 10% povidone-iodine and draped.

An incision was made along the inguinal crease and extended obliquely down the midline. Hindlimb dissection began medially, dividing medial thigh muscles and muscular branches from the pelvis. The limb was hyper-adducted and rotated to expose the posterior surface, and the skin incision was completed circumferentially. Posterior thigh muscles were divided near their origins along the sacrum, ilium, and ischium. The sciatic nerve was transected, and dissection converged medially, preserving the femoral vascular pedicle, the iliopsoas, and the femoral joint.

The femoral vein, artery, and nerve were dissected 2–3 cm above the inguinal ligament. A skin flap was raised to access the foot extensor muscles for distal sampling. After baseline samples, the femoral pedicle was divided—marking the start of ischemia—and the iliopsoas was divided with subsequent femoral head dislocation to complete the amputation. Immediately after, the femoral artery was cannulated with a 14G angiocatheter (BD Angiocath™, USA) and flushed with 1L cold Low Potassium Dextran Solution (LPD, Servator-P, Global Transplant Solutions, Canada), an extracellular preservation solution with high Na+ (138 mmol/L) and low K+ (6 mmol/L) with Dextran-40 at 50 g/L concentration (28). The detailed composition is in Supplementary Table S1. Limb weight was recorded, after which the limb was wrapped in LPD-soaked gauze, placed in a sealed bag, and set on ice. It was then immediately transported to a 4 °C cold room for static cold storage.

For the perfusion group, after the same surgical steps and before pedicle division, a midline laparotomy exposed the inferior vena cava, which was cannulated (DLP 18F Single Stage Venous Cannula, Medtronic, USA). The animal was exsanguinated, marking ischemia onset. Blood was collected into a 40 μm filtered reservoir (ATF 40 Fast Start Kit, Fresenius Kabi, Germany), washed with an autotransfusion system (Fresenius Kabi C.A.T.S®plus Continuous Auto Transfusion System), and recollected into a sterile bag. Hematocrit was measured, and the limb was transported to the perfusion room on ice after 1 L cold LPD flush for *ex vivo* perfusion.

### 2.3 *Ex vivo* perfusion system

#### 2.3.1 Perfusion system setup

The *ex vivo* perfusion system, modeled after the Toronto *ex vivo* Lung Perfusion system (14), included an oxygenator with reservoir (Eurosets RemoweLL2 Oxygenator/Reservoir, Nipro Canada), peristaltic pump (Masterflex® L/S® Digital Drive with Easy-Load® 3 Pump Head, 115/230 VAC), heater-cooler (CSZ Hemotherm 400MR, USA), gas tank (95% O₂/5% CO₂) with flow meter, and a custom limb receptacle with passive venous return. Additional components were a temperature probe (Traceable, Panel-Mount Thermometer with Calibration, USA) and a patient monitor (General Electric Transport Pro, USA). Components were connected using sterile silicone tubing (Masterflex® L/S® Precision Pump Tubing, Platinum-Cured Silicone, 3/8″ and 1/4″) as shown in Figure 1. Perfusate was pumped from the reservoir to the oxygenator, then through the limb, with venous outflow returning to the reservoir. Two pressure transducers monitored the inline and compartment pressures.

#### 2.3.2 Perfusion procedure and monitoring

The circuit was primed with 3 L of Low Potassium Dextran solution enriched with bovine serum albumin (Millipore Sigma, USA) at 2.5 g/dl (Oncotic properties represented in Supplementary File, Figure 4). Additives included 5,000 IU heparin (Heparin Leo 1,000 IU/ml), 500 mg methylprednisolone (Solu-Medrol, Pfizer, USA), L-alanine L-glutamine (GlutaMAX, Gibco, Thermo Fisher Scientific, USA) at 2 mm, 30 ml 8.4% NaHCO₃ (1M) (Pfizer, USA), 3 ml 50% dextrose (Pfizer, USA), and 0.1 U/kg regular insulin (Humulin-R, Lilly, USA). Autologous washed RBC concentrate was added to achieve a hematocrit 10%–15% (21, 29), based on baseline hematocrit. The remaining RBC concentrate was stored at 4 °C and used for three equal exchanges at 6, 12, and 18 h.

Hourly perfusate samples were taken from arterial inflow and venous outflow and analyzed with a blood gas analyzer (GEM Premier 5000, Instrumentation Laboratory, Bedford, USA). L-alanyl-L-glutamine was infused at 2 ml/h; 8.4% (1 M) NaHCO₃ was added to maintain pH >7.2. 50% dextrose, with or without 0.1 U/kg insulin, was used to keep glucose between 3.6–7 mmol/L, with insulin given at exchanges and when glucose exceeded 7 mmol/L. Every four hours, additional perfusate samples were tested for free hemoglobin.

Lactate, K^+^ and Ca^+2^ productions (mmol/min/kg tissue) were calculated as [(Venous Solute – Arterial Solute concentration) × Flow]/(1,000 × Initial Limb weight); where solute concentrations are in mmol/L, flow is in ml/min, limb weight in kg. Oxygen consumption (ml O_2_/min/kg tissue) was calculated for every 6 h as (arterial oxygen content - venous oxygen content) × flow/(100xinitial limb weight in kg). Arterial and venous oxygen content were derived from blood gas measurements using the equation 1.39 × Hb × Fraction of O_2_Hb + 0.0031 × pO_2_ (24). Glucose uptake was calculated as (Arterial Glucose- Venous Glucose) × Flow)/(1,000 × Initial Limb weight) where glucose concentration is mmol/L, flow in ml/min, limb weight in kg.

Inflow pressure was maintained at 30 mmHg for the first 15 min, then increased to 60–65 mmHg within an hour by adjusting pump speed. Peripheral vascular resistance was calculated by first correcting total system pressure for the additional pressure generated by the arterial cannula, using the circuit-only pressure–flow calibration curve (Figure 3, Supplementary File) to obtain organ (vascular) pressure at each flow rate. Vascular resistance was then derived using Ohm's law (ΔPressure = Flow × Resistance), assuming venous pressure to be zero because of the open venous drainage. Normalized flow rates were calculated by dividing the flow at each time point by the initial limb weight in kg. Muscle temperature was continuously monitored and kept at 28 °C–32 °C by gradually warming the perfusate with the heater-cooler. pO₂ was maintained between 200 and 400 mmHg.

To measure compartment pressure, a stab incision was made 1 cm lateral to the mid-tibial border. An 18G side-ported needle (Hamilton™ Metal Hub Side Port Needles, Fisher Scientific, USA), primed with saline, was inserted into the anterior compartment and left in place, with the transducer levelled at the needle tip (30). The system was flushed and zeroed before hourly readings.

**Figure 2:**
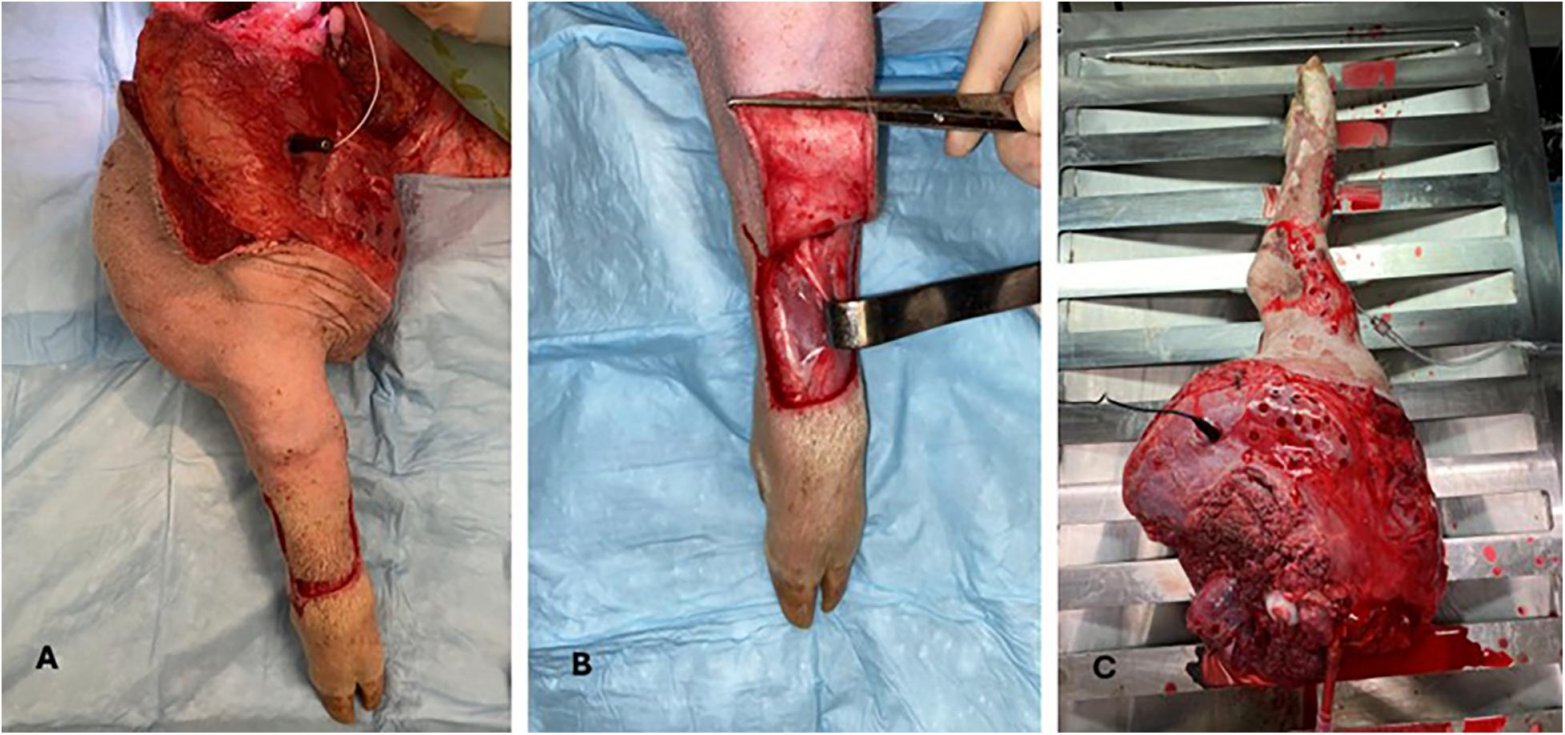
Porcine total hindlimb model **(A)** intraoperative view, exposed muscles of the thigh were sampled as proximal muscle samples, **(B)** A skin flap was elevated off the foot to expose the distal muscle biopsy site **(C)** hindlimb during *ex vivo* perfusion with temperature and compartment pressure probes in place.

Tissue samples from the femoral artery, vein, and nerve were taken at procurement and at the end of the experiment. Muscle samples were collected every six hours from the anteromedial thigh (proximal) and foot (distal) (Figure 2). Skin samples were obtained every six hours from the anteromedial tibia.

At the end of perfusion or cold storage, limb weights were recorded, and the weight change was calculated as the percentage difference from baseline. Neuromuscular stimulation was performed at the femoral nerve using a TENS device (TENS 7000, Carex, China) at 3 Hz and 250 μs (31). To confirm circulation, 0.1 mg/kg ICG (2.5 mg/ml) was injected into the arterial line, and near-infrared imaging (700–900 nm) was done using a Spy-Phi camera (Stryker, USA).

### 2.4 Sample handling and analyses

Vein, artery, nerve, skin, and muscle samples were fixed in 10% formalin, paraffin-embedded, and sectioned at 5 μm for H&E staining. Histological slides were digitized using an Aperio ScanScope slide scanner (Leica Biosystems, Vista, USA) at ×20 objective magnification. Digital images were reviewed using Aperio ImageScope (Vista, USA) software. Muscle morphology was evaluated over time by two blinded reviewers using a modified Histology Injury Severity Score (HISS) for myocyte injury (32) (Supplementary Table S2).

Additional 5 μm sections were cut for baseline endpoint muscle samples for CD31 immunostaining. Briefly, the sections were deparaffinized. Antigen retrieval was performed at 120 °C using a 1:100 dilution of antigen retrieval buffer (Abcam, ab93678, Cambridge, UK). Blocking was achieved by incubation with 5% BSA. The primary antibody (Abcam, ab28364, Cambridge, UK) was applied overnight at a 1:100 dilution. After inactivating endogenous peroxidases with 3% hydrogen peroxide in methanol, the secondary antibody (Abcam, ab288151, Cambridge, UK) was applied at a 1:500 dilution. DAB-Peroxidase reaction was performed with (Abcam, ab64238), and slides were counterstained with Hematoxylin.

Additional muscle samples were snap-frozen in liquid nitrogen for ATP quantification and stored at −80 °C. ATP was measured using the ATP Assay Kit (Abcam, ab83355, Cambridge, UK) according to the manufacturer's protocol. Ten mg of tissue was homogenized in 2N perchloric acid for deproteinization, neutralized with 1M potassium hydroxide to pH 6.5–8, and analyzed colorimetrically (SpectraMax-Plus, Molecular Devices, USA).

Perfusate samples for free hemoglobin measurement were centrifuged at 3,000 g for 5 min, and the supernatant was snap-frozen in liquid nitrogen. After thawing, the absorbance spectrum (500–700 nm) was measured using the SpectraMax-Plus device, and concentrations were calculated from a standard calibration curve.

### 2.5 Statistical analyses

Data presented as mean ± SD. Baseline and endpoint comparisons for electrolytes, limb weight, compartment pressure, flow, and resistance were assessed for normality. Normally distributed data were analyzed using paired *t*-tests; non-normally distributed data were analyzed using Wilcoxon matched-pairs tests. For ATP and HISS scores, normality was tested, and repeated-measures ANOVA with Tukey's test was used; additional *post hoc* unpaired *t*-tests compared shared time points. Analyses were performed with GraphPad Prism 10.3.0 (Dotmatics, San Diego, USA).

## 3 Results

The animals in the 24-h *ex vivo* perfusion group had an average weight of 39.38 ± 1.6 kg. The procured limbs weighed 4.06 ± 0.22 kg, whole blood collection totalled 1.34 ± 0.22 L and the washed RBC volume was 1.1 L ± 0.1. The mean warm ischemia time was 11.8 ± 2.64 min, the mean cold ischemia time was 56.2 ± 7.19 min, and the total ischemia time was 68.00 ± 9.7 min. Time from pedicle division—including cold flush, limb handling, and documentation—to transfer of SCS limbs on ice to the 4 °C cold room was 17 ± 2.34 min (Table 1).

**Table 1:** General information from the 24-h perfusion experiments.

Pig Nu	Pig weight	Limb weight	Whole blood volume	pRBC volume	Warm ischemia time	Cold ischemia time	Total ischemia time
1	39	4.1	1.4	1.1	10	55	65
2	39	3.9	1.5	1.3	9	51	60
3	37.1	3.93	1.3	1.1	10	55	65
4	41	3.95	1.2	0.9	15	70	85
5	40.8	4.43	1.3	1.1	15	50	65
Mean (SD)	39.38 (1.59)	4.06 (0.22)	1.34 (0.11)	11 (0.14)	11.8 (2.64)	56.2 (7.19)	68 (9.74)

SD, standard deviation.

Weights are reported in kilograms (kg), volumes in litres (L), and time in minutes. Values are presented as mean and standard deviation (SD).

### 3.1 Maintained temperature and peripheral resistance in 24-h perfusion

Muscle temperature at baseline was 26.2 ± 2.2 °C, reaching an average peak of 30.1 ± 0.7 °C at 12 h and 29.0 ± 1.5 °C at the endpoint.

**Figure 3:**
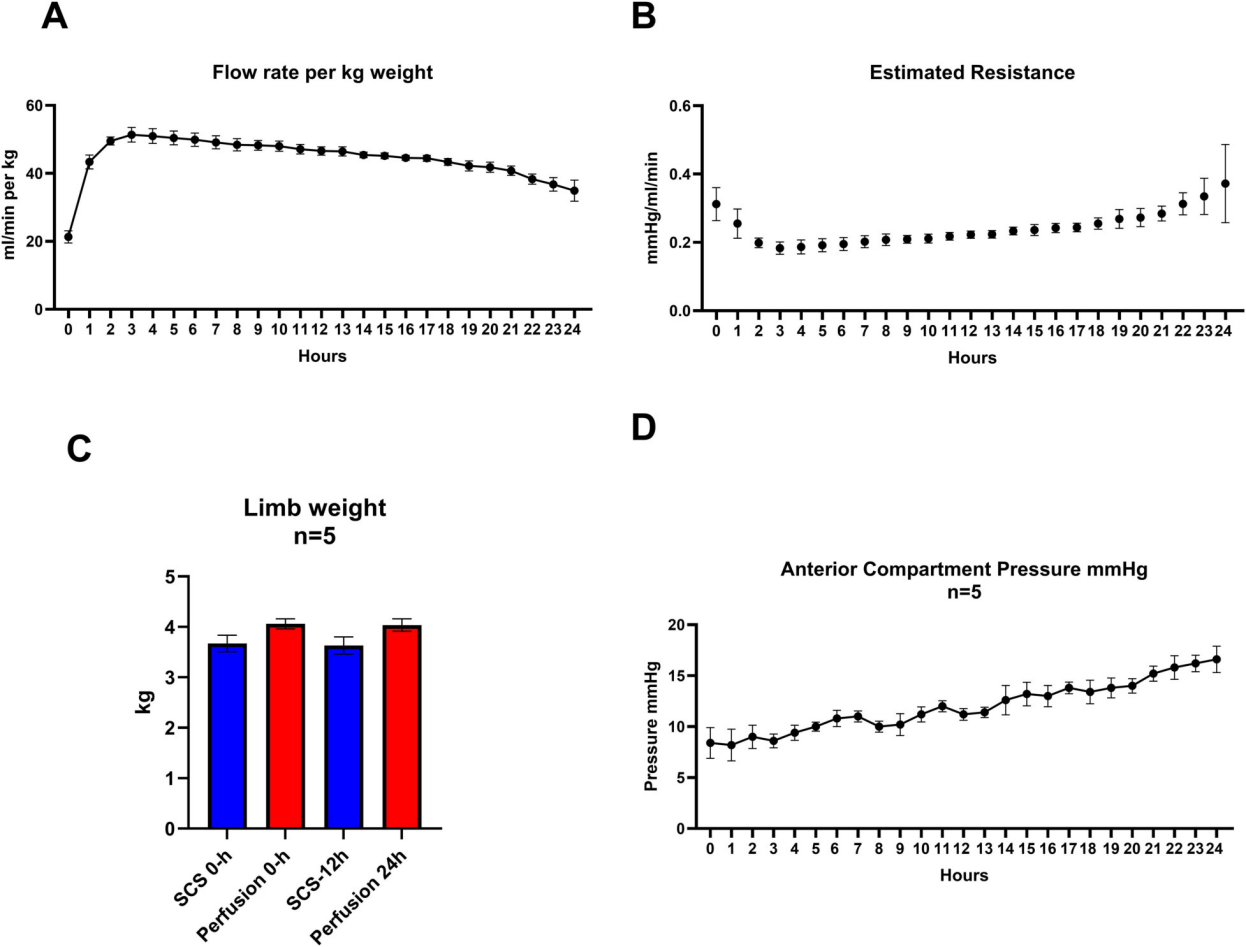
Perfusion metrics **(A)** flow per kg tissue over time, error bars represent SEM **(B)** estimated resistance over time, error bars represent SEM **(C)** weight change in cold storage controls and perfusion groups, error bars represent SEM, weight difference during the experiment was not significant in both groups (SCS *p* = 0.056), perfusion *p* = 0.63), Wilcoxon and paired *t*-test results respectively) **(D)** change in anterior compartment pressure over time 8.4 ± 3.4 mmHg at baseline to 16.6 ± 2.9 mmHg at the endpoint (*p* = 0.036). Error Bars represent SEM.

After achieving the target perfusion pressure during the first hour, the resulting flow per kg of weight was 43.4 ± 4.6 ml/min. Pump speed was subsequently adjusted to maintain this pressure, leading to a peak flow at 3 h (51.4 ± 4.8 ml/min). Flow then declined to 34.92 ± 6.9 ml/min at 24 h (difference between peak flow and endpoint, *p* = 0.01). Calculated resistance at the start of perfusion was 0.31 ± 0.05 mmHg/ml/min, reached a nadir at 3 h (0.19 ± 0.02 mmHg/ml/min), and gradually increased to 0.38 ± 0.11 mmHg/ml/min at the endpoint. There was no statistically significant difference between baseline and endpoint (*p* = 0.41) or between the lowest and endpoint resistance (*p* = 0.06). Figures 3A,B show changes in flow per kg tissue and resistance over time.

### 3.2 24-h perfusion did not show a significant weight change

The mean baseline weight of cold storage controls was 3.671 ± 0.4 kg, while perfusion limbs weighed 4.060 ± 0.2 kg. After endpoint, the mean weights were 3.632 ± 0.4 kg and 4.036 ± 0.3 kg, respectively. Mean percent weight change was −1.08 ± 1.2% in controls and −0.53% ± 5.64 in the perfusion group. Neither group showed a significant change from baseline, and there was no statistically significant difference between the groups at either time point (Figure 3). The mean anterior compartment pressure in the perfusion group increased from 8.4 ± 3.4 mmHg at baseline to 16.6 ± 2.9 mmHg at the endpoint (*p* = 0.036). Figures 3C,D show the change in weight and in anterior compartment pressure over time.

### 3.3 Stable pH, K^+^, glucose uptake and increased lactate, Na^+^ and Ca^+2^ in *ex vivo* perfusion

**Figure 4:**
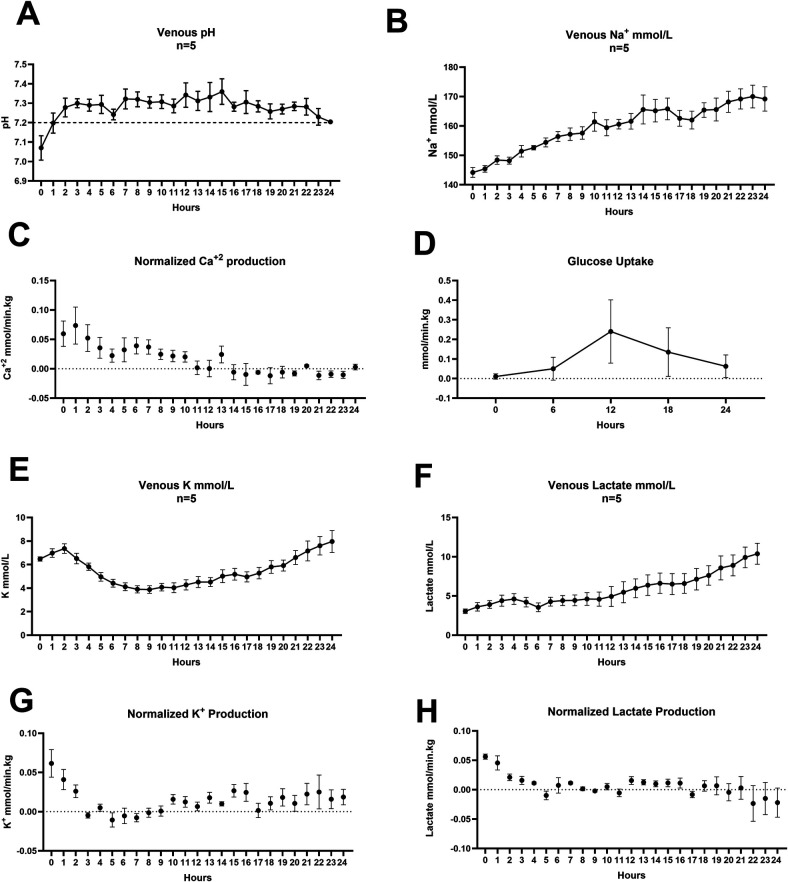
Perfusate analyses of electrolytes, glucose and lactate. **(A)** 93 ± 12.41 ml Sodium Bicarbonate was added on average to maintain pH **(B)** Sodium increased from 144.2 ± 3.8 to 169.2 ± 9.3 mmol/L. **(C)** Ca^+2^ production was reduced from 0.06 ± 0.05 mmol/min.kg to 0.003 ± 0.01 mmol/min.kg **(D)** Glucose uptake rate increased in the first 12-h from 0.01 ± 0.03 mmol/min.kg at baseline to 0.24 ± 0.36 mmol/min.kg, then decreased to 0.06 ± 0.13 mmol/min.kg No differences were seen between groups 0–12 h (*p* = 0.9), 12–24 h (*p* = 0.9), Dunn's multiple comparisons test. 73.20 ± 3.50 ml 50% dextrose was added on average to maintain glucose levels in the physiological range, **(E)** Potassium increased in the first two hours then reached a nadir 3.9 ± 0.7 mmol/L at 9 h, gradually increasing to 7.9 ± 2.1 mmol/L by 24-h **(F)** Lactate accumulated in the system rising from 3.1 ± 0.6 at baseline to 10.38 ± 2.9 mmol/L at endpoint **(G)** Potassium production normalized to weight and flow gradually decreased from 0.06 ± 0.04 mmol/min.kg to 0.02 ± 0.02 at 24-h **(H)** Lactate production similarly decreased during the perfusion from 0.06 ± 0.01 mmol/min.kg to −0.02 ± 0.05. Error bars represent SEM.

**Figure 5:**
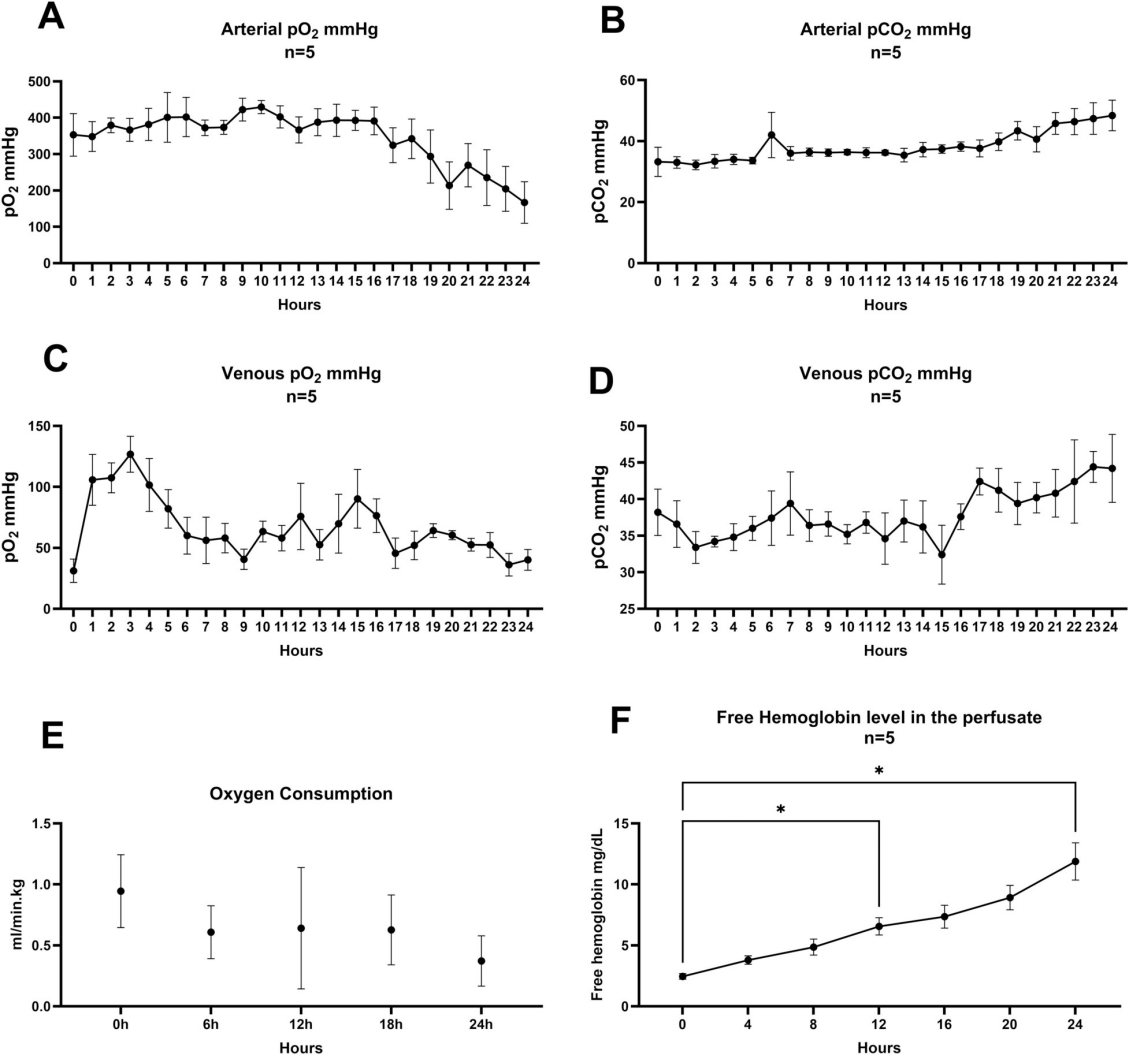
Perfusate gases and free hemoglobin during the perfusion. **(A)** Arterial pO_2_ was 353 ± 131 mmHg at baseline and 166.8 ± 127.8 at 24-h **(B)** Arterial pCO_2_ was 33.2 ± 10.64 mmHg at baseline and 48.4 ± 11.26 mmHg at 24-h **(C)** Venous pO_2_ was 31.2 ± 21.4 mmHg at baseline and 40.2 ± 18.95 mmHg at 24-h **(D)** Venous pCO_2_ was 38.2 ± 7.1 mmHg at baseline and 44.2 ± 10.4 mmHg at 24-h **(E)** Oxygen consumption was highest at baseline 0.94 ± 0.3 ml/min.kg then declined to 0.4 ± 0.18 ml/min.kg, there were no differences in any of the 6 h marks. (0–24 h *p* = 0.0577, Tukey's Multiple comparisons test repeated measures one way ANOVA. **(F)** Increase in free hemoglobin was seen* 0 h–12 h, *p* = 0.0460, *0 h–24 h *p* = 0.0311 values in s Tukey's multiple comparisons test for repeated measures one way ANOVA, Error bars represent SEM.

We were able to maintain pH, arterial and venous pO_2_ and CO_2_ levels, as well as K^+^. These parameters did not show significant changes from baseline values during 24-h *ex vivo* perfusion. Lactate (*p* = 0.002), Na^+^ (*p* = 0.006) and Ca^+2^ (*p* < 0.001) accumulated over time in the perfusion system. Table 2 shows baseline and endpoint biochemical characteristics of the perfusion experiments. However, the normalized production of Ca^2^, K, and lactate showed a decreasing trend, suggesting stabilization of metabolic homeostasis during the latter phases of perfusion. Glucose uptake peaked during the first 12 h of perfusion at 0.24 ± 0.36 mmol/min.kg, then gradually declined to 0.06 ± 0.13 mmol/min.kg by 24 h, there were no differences between the rates. Figure 4 shows the changes in biochemical parameters over time. Arterial pO_2_ declined over time; however, it remained within the target 200–400 mmHg range. Venous pO_2_ followed the pattern of arterial pO_2_. Oxygen uptake was calculated to be highest at baseline, 0.94 ± 0.3 ml/min.kg gradually decreasing to 0.40 ± 0.18 ml/min.kg. No changes were detected. Figure 5 shows the perfusate gases (A, B, C and D) and oxygen uptake over time.

**Table 2:** Comparisons of baseline and endpoint measurements in perfusate analysis.

Parameter	Baseline		Endpoint		*p*
	Mean (SD)	Median (IQR)	Mean (SD)	Median (IQR)	
pH venous	7.07 (0.14)	7.010 (0.18)	7.204 (0.005)	7.200 (0.01)	0.1250*
Na^+^ venous mmol/L	144.2 (3.76)	144.0 (6.50)	169.2 (9.31)	167 (16.5)	0.0060
K^+^ venous mmol/L	6.48 (0.40)	6.600 (0.8)	7.96 (2.08)	8.30 (3.15)	0.2379
Ca^+2^ venous mmol/L	0.59 (0.16)	0.6 (0.31)	1.26 (0.10)	1.25 (0.19)	0.0003
Lactate venous mmol/L	3.06 (0.63)	2.70 (1.2)	10.38 (2.99)	10.60 (5.95)	0.0027
Glucose venous mmol/L	3.84 (0.52)	3.60 (1.0)	4.60 (3.06)	3.50 (4.15)	0.8125*
pO_2_ arterial mmHg	353 (131.0)	407 (212.0)	166.8 (127.8)	110 (185.0)	0.0625*
pO_2_ venous mmHg	31.2 (21.39)	44.0 (40.0)	40.20 (18.95)	32.00 (37.50)	0.2629
pCO_2_ arterial mmHg	33.20 (10.64)	36.00 (19.00)	48.40 (11.26)	43.00 (18.50)	0.1126
pCO_2_ venous mmHg	38.20 (7.08)	42.00 (13.50)	44.20 (10.38)	46 (15.50)	0.3750*
Ca^+2^ production	0.06 (0.05)	0.06 (0.1)	0.002 (0.01)	0 (0.02)	0.0480
Lactate production	0.06 (0.01)	0.06 (0.03)	−0.02 (0.06)	−0.09 (0.13)	0.0433
K^+^ production	0.06 (0.04)	0.04 (0.1)	0.02 (0.02)	0.03 (0.05)	0.111
Glucose uptake	0.01 (0.04)	0.01 (0.07)	0.05 (0.13)	−0.01 (0.31)	0.9**
O_2_ uptake	0.94 (0.3)	1.02 (0.6)	0.37 (0.22)	0.4 (0.2)	0.058***

All other *p*-values were from paired *t*-tests. Calcium, lactate, potassium production, and glucose uptake are in mmol/kg/min; oxygen uptake is in ml/kg/min.

* Wilcoxon matched-pairs test.

** Kruskal–Wallis.

*** ANOVA.

### 3.4 Increased free hemoglobin in the perfusate starting at the 12th hour of perfusion

Mean free hemoglobin at baseline was 2.5 ± 0.5 mg/dl. At 12 h, it increased significantly to 6.6 ± 1.6 mg/dl (*p* = 0.0460). By 24 h, free hemoglobin levels further increased to 11.9 ± 3.4 mg/dl (*p* = 0.0311) compared to baseline (Figure 5).

### 3.5 24-h perfusion showed stable ATP levels in both biopsy sites

In the cold-storage control group, ATP levels in both proximal and distal muscle samples declined significantly after 12 h relative to baseline; at 6 h, the decrease was not significant. In the proximal muscle, ATP levels decreased from 0.50 ± 0.1 nmol/μl at baseline to 0.27 ± 0.1 mmol/L at 6 h (*p* = 0.09) and to 0.15 nmol/μl ± at 12 h (*p* = 0.009). In the distal muscle, ATP levels decreased from 0.34 ± 0.2 nmol/μl at baseline to 0.2 ± 0.1 mmol/L at 6 h (*p* = 0.09) and to 0.08 ± 0.08 ± 0.1 nmol/μl at 12 h (*p* = 0.021).

**Figure 6:**
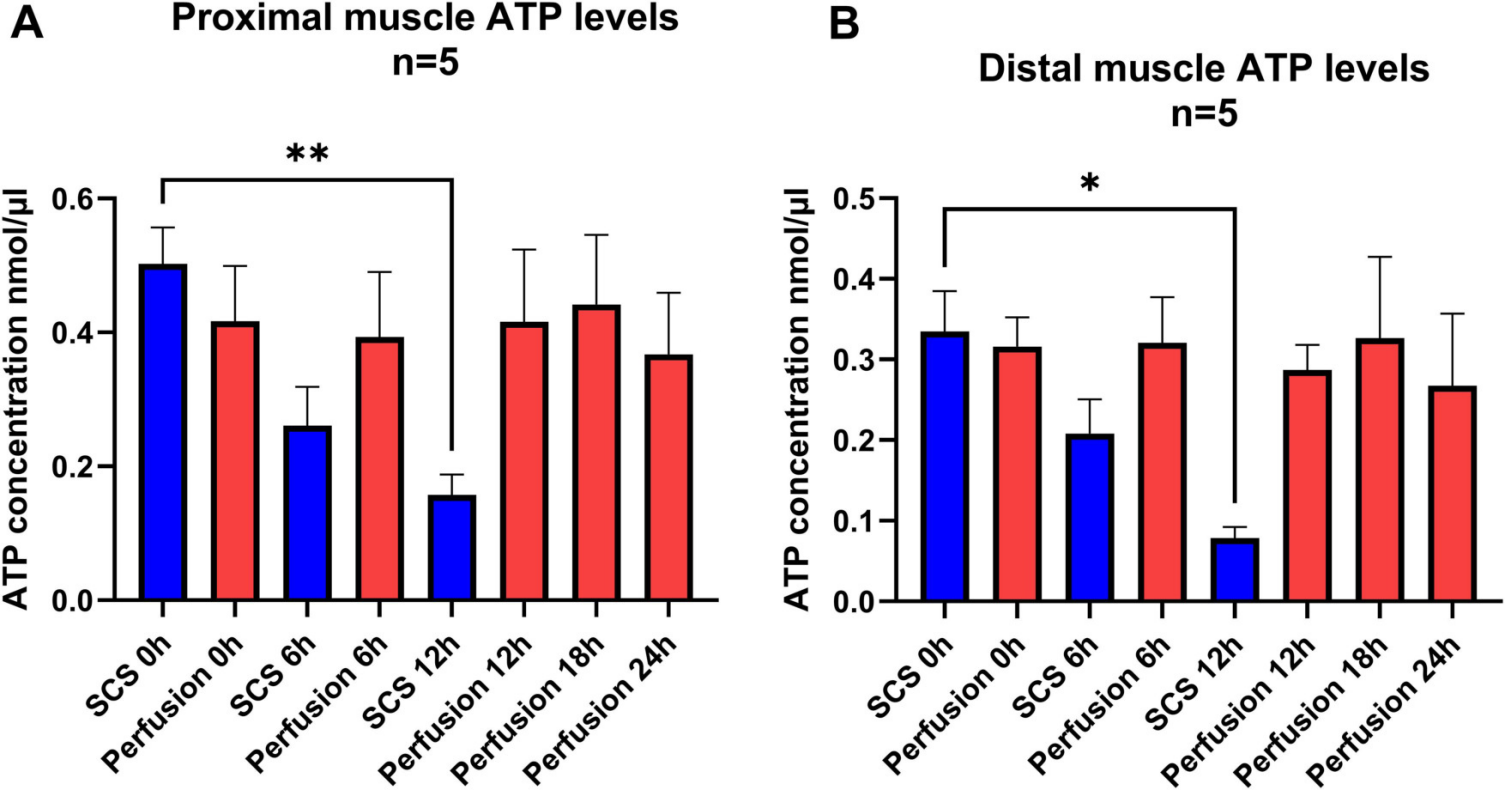
ATP levels in muscle samples **(A)** proximal muscle, in static cold storage the difference was not significant at 6 h (*p* = 0.09) but significant at 12-h (**p = 0.009), in perfusion group, there was no significant change at 24 h (*p* = 0.1727) in comparison to baselines. *P* values represent result of Tukey's multiple comparisons test, repeated measures one way ANOVA. *Post hoc* multiple unpaired *t*-test with Holm-Sidak correction for shared time points showed no difference in 0-h (*p* = 0.4753), 6-h (*p* = 0.4753) and 12-h (*p* = 0.1441) **(B)** Distal muscle, in static cold storage the difference was not significant at 6 h (*p* = 0.09) but significant at 12-h (*p = 0.021), In perfusion group, there was no significant change at 24 h (*p* = 0.9759) *P* values represent result of Tukey's multiple comparisons test, repeated measures one way ANOVA. *Post hoc* multiple unpaired *t*-test with Holm-Sidak correction for shared time points showed no difference in 0-h (*p* = 0.7789) and 6-h (*p* = 0.2757) at 12-h difference was significant (*p* = 0.0008) Error bars represent SEM in both graphs, 10 mg of muscle tissue were used for measurements.

In the perfusion group, ATP levels at 24 h did not decrease significantly from baseline. In the proximal muscle, ATP levels were 0.42 nmol/μl ± 0.2at baseline and 0.37 nmol/μl (SD: 0.20) at 24 h (*p* = 0.17). In the distal muscle, ATP levels were 0.32 ± 0.1 nmol/μl at baseline and 0.27 nmol/μl at 24 h (*p* = 0.97) (Figure 6).

### 3.6 Conserved muscle morphology after 24 h of *ex vivo* perfusion

**Figure 7:**
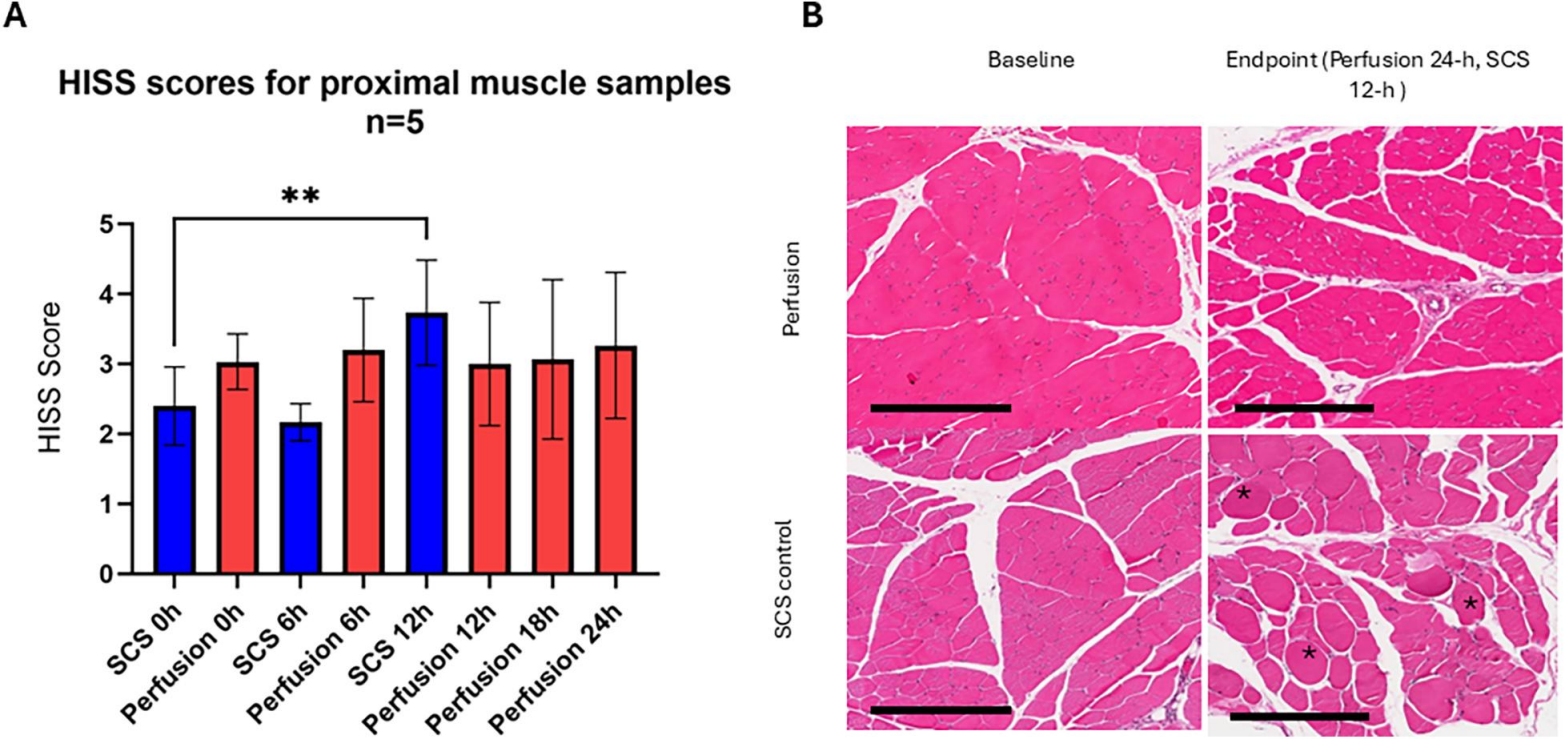
Muscle morphology in proximal muscle samples. **(A)** Total Histology Injury severity scores, in static cold storage the difference was not significant at 6 h (*p* = 0.7362) but significant at 12-h (**p = 0.008), In perfusion group, there was no significant change at 24 h (*p* = 0.99) in comparison to baseline, *P*-values represent result of Tukey's multiple comparisons test, repeated measures one way ANOVA. *Post hoc* multiple unpaired *t*-test with Holm-Sidak correction for shared time points showed no difference in 0-h (*p* = 0.1411), 6-h (*p* = 0.0548) and 12-h (*p* = 0.1946). Error Bars represent SEM. **(B)** Representative images from proximal muscle biopsies, H&E staining, 10×. Asterisk: Hypoxic fibers, HISS, histology injury severity. Scale bar: 300 μm.

The baseline total muscle injury score in the proximal muscle for the perfusion group was 3.03 ± 0.4, and for the SCS group, it was 2.40 ± 0.6. At the 24 h endpoint, the scores were 3.26 ± 1.0 and 3.73 ± 0.8, respectively. In the cold storage controls, the increase was significant compared to baseline (*p* = 0.008). In the perfusion group, no significant difference was noted (*p* = 0.99) (Figure 7).

**Figure 8:**
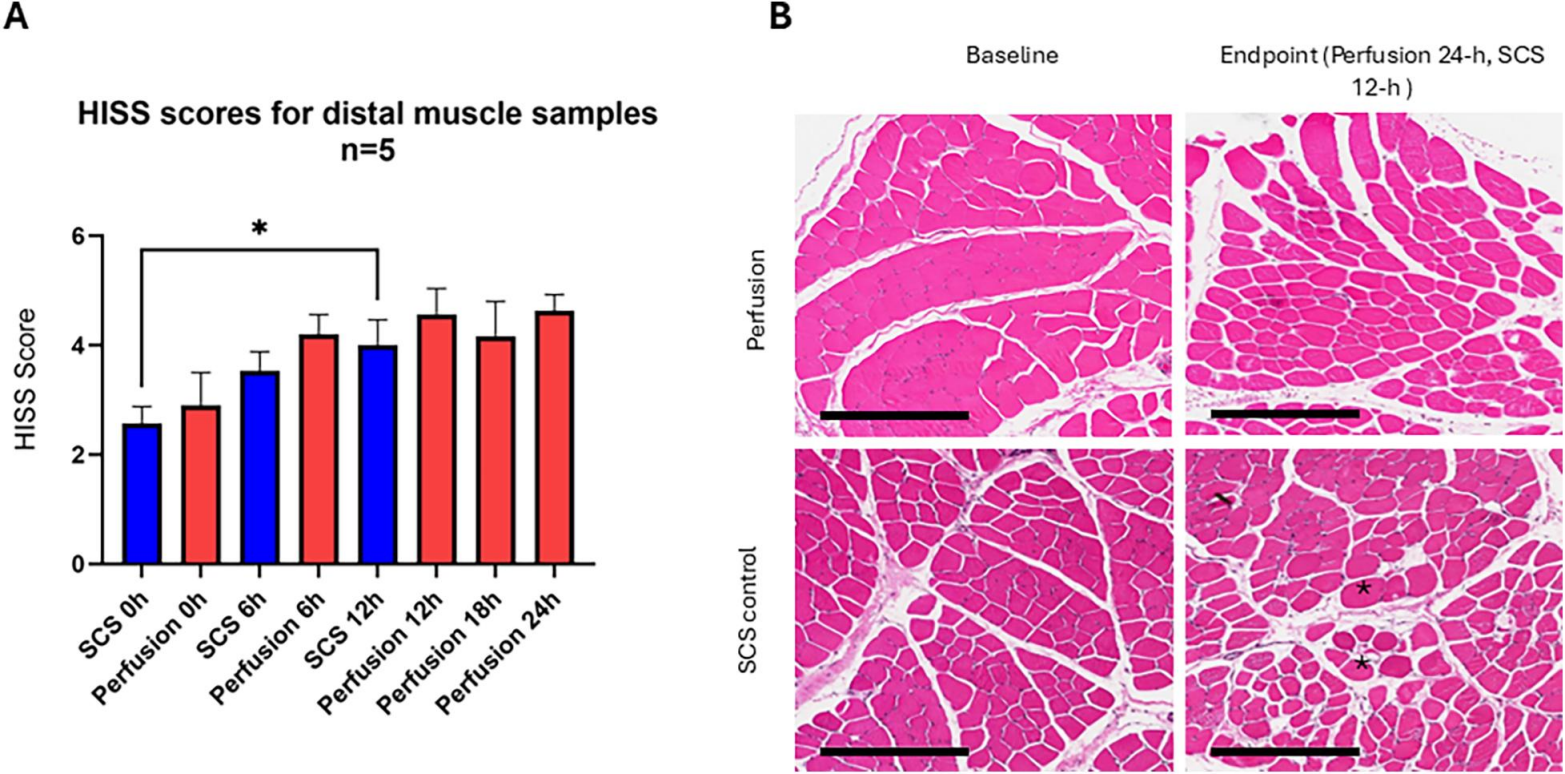
Muscle morphology in distal muscle samples. **(A)** Total Histology Injury severity scores, in static cold storage the difference was not significant at 6 h (*p* = 0.3493) but significant at 12-h (*p = 0.03), In perfusion group, there was no significant change at 24 h (*p* = 0.3533) in comparison to baselines, *P*-values represent result of Tukey's multiple comparisons test, repeated measures one way ANOVA. *Post hoc* multiple unpaired *t*-test with Holm-Sidak correction for shared time points showed no difference in 0-h (*p* = 0.6629), 6-h (*p* = 0.5314) and 12-h (*p* = 0.6629). Error Bars represent SEM. **(B)** Representative images from proximal muscle biopsies, H&E staining, 10×. Asterisk, hypoxic fibers; HISS, histology injury severity. Scale bar: 300 μm.

**Figure 9:**
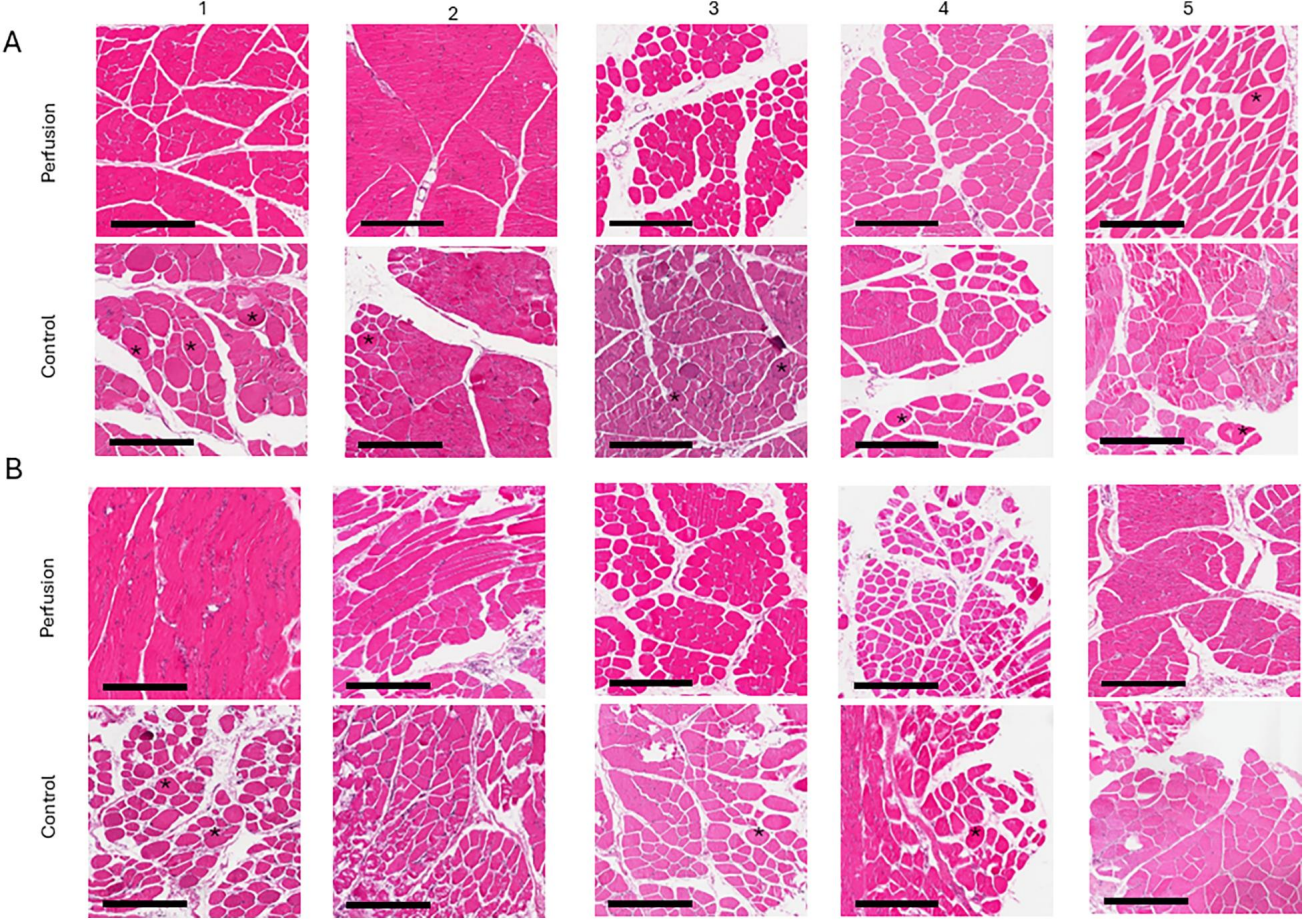
24-h perfusion (*n* = 5) and 12-h cold storage (*n* = 5) representative images, H&E staining, 10× **(A)** proximal muscle: perfusion group showed moderate interstitial edema in images 3, 4 and 5 with variability in myocytes and some hypoxic fibers (asterisk). Images 1 and 2 did not show edema, variation or damage. In controls, images 1,2,3,4 and 5 shows hypoxic myocytes (Asterisk) and variation. **(B)** Distal muscle: Perfusion group, images 2,3,4 show moderate interstitial edema and variation. In controls image 1, 3, and 4 show variation and hypoxic fibers (Asterisk), in image 2 a cluster of myocytes with damaged borders are seen on the bottom left corner of the image. Scale bar: 300 μm.

Similarly, the baseline total injury score in the distal muscles for the perfusion group was 2.90 ± 1.3, and for the SCS group, it was 2.56 ± 0.7. At the endpoint, the scores were 4.63 ± 0.7 and 4.00 ± 1, respectively. In the cold storage controls, the increase was significant (*p* = 0.03). In the perfusion group, no significant difference was detected (*p* = 0.35) (Figure 8). Breakdown of scoring elements of interstitial edema, variation in myocytes and damaged myocytes is presented in Supplementary Table S3. Additional 24 h endpoint H&E images from the five different perfusion experiments, along with five different controls, are shown in Figure 9.

Representative images from H&E sections for skin, femoral nerve, artery and vein samples are shown in the Supplementary File. No apparent morphological differences were detected.

**Figure 10:**
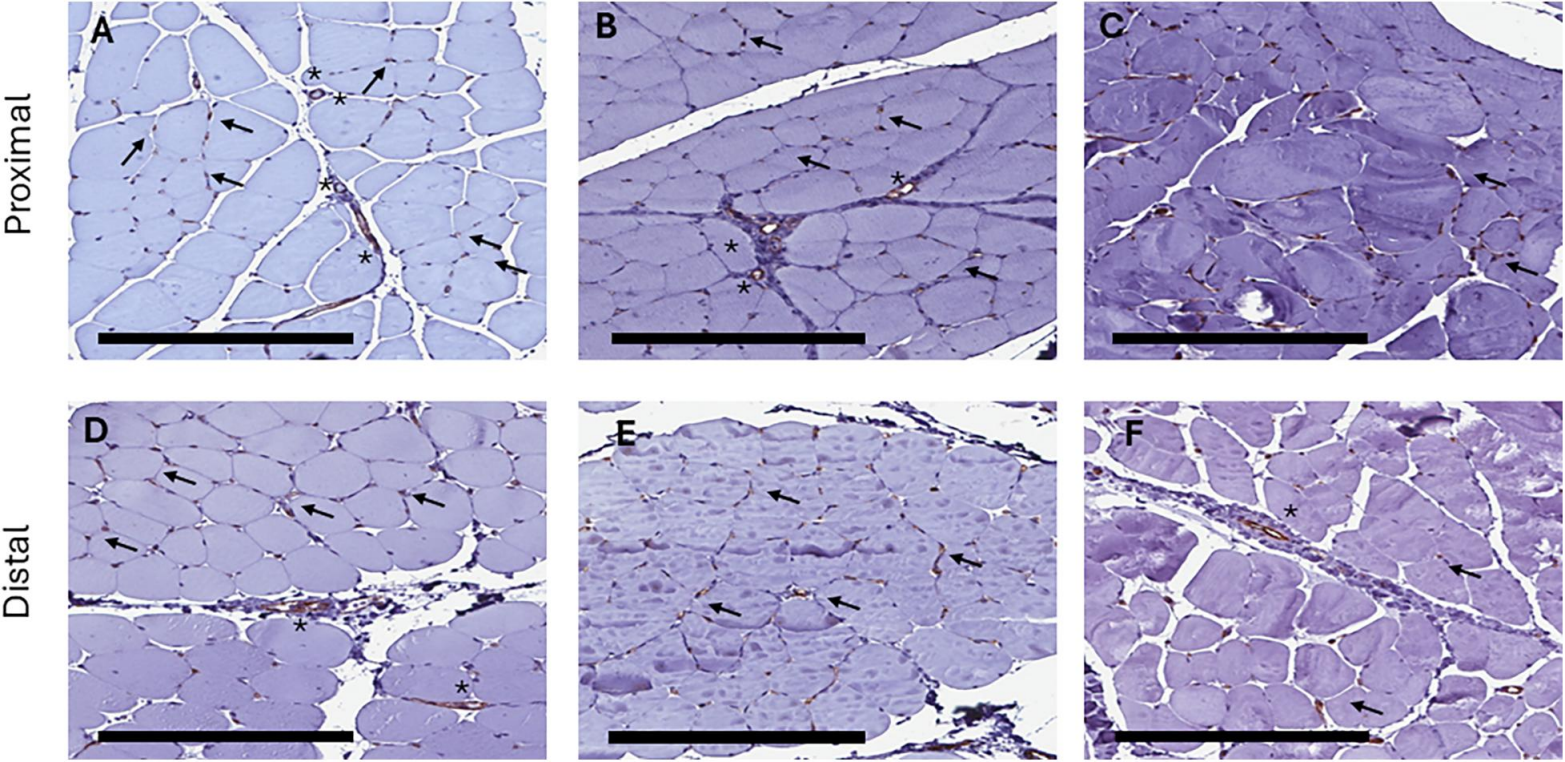
CD-31 immunostaining in proximal and distal muscle samples at baseline and 24-h perfusion or 12-h cold storage, 20× baseline characteristics **(A)** of CD-31 immunostaining show dense CD-31 positive capillaries (arrows) and arterioles/venules (asterisk). After 24-h *ex vivo* perfusion **(B)** baseline CD-31 staining properties were retained. 12-h cold storage controls **(C)** also show CD-31 positive microvasculature. Similar staining properties were seen in distal muscle samples at baseline **(D)**, 24-h perfusion **(E)** and 12-h cold storage **(F)**.

CD-31 staining showed capillaries surrounding the fibres and larger arterioles and venules in the perimysium at baseline. The staining properties were retained after 24-h *ex vivo* perfusion, suggesting intact microvasculature without endothelial shear. In control groups, arterioles and venules retained CD-31 positive staining properties; however, capillary visualization was harder (Figure 10).

### 3.7 ICG injection and neuromuscular stimulation

**Figure 11:**
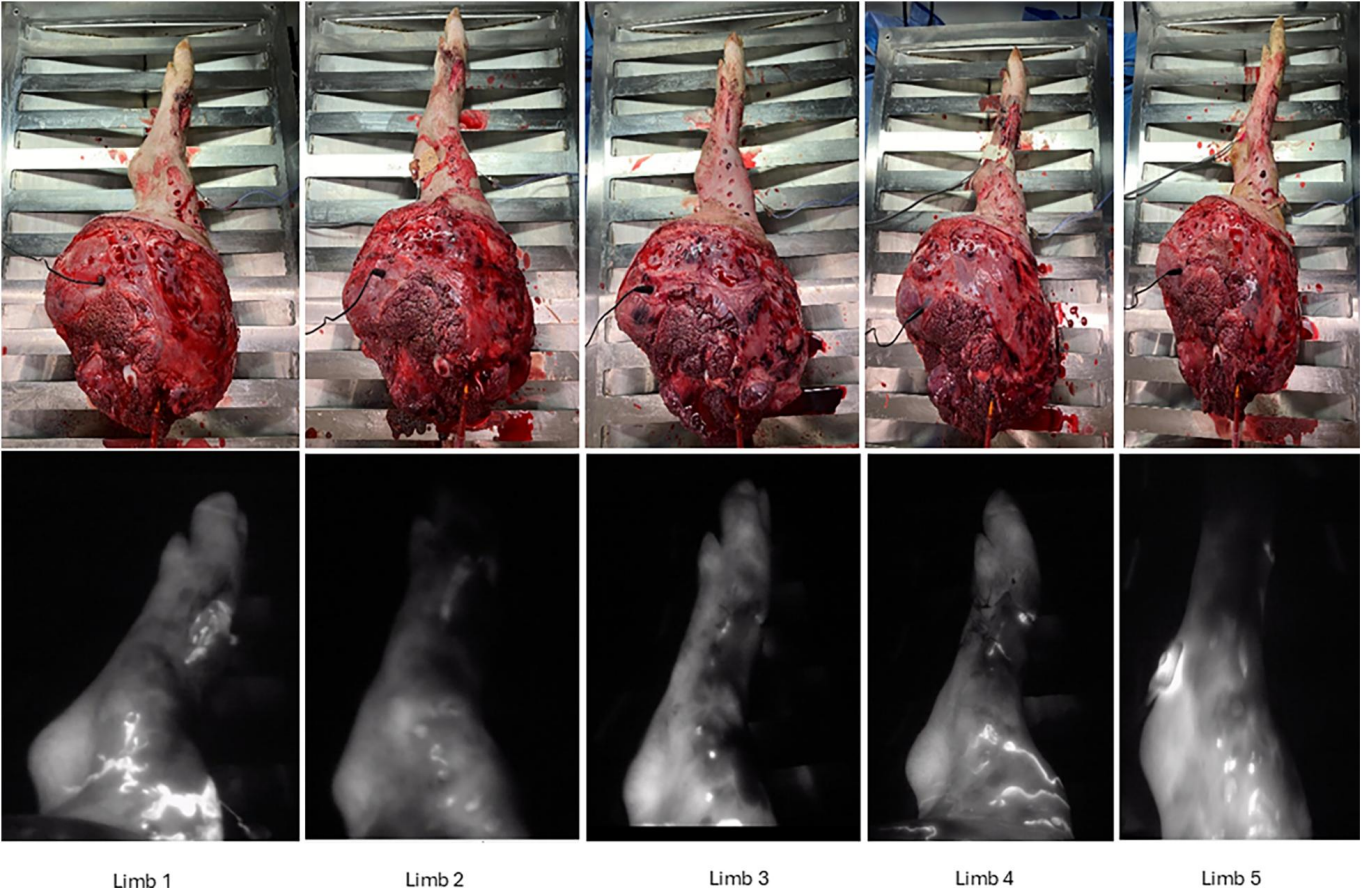
Images of perfused limbs after 24 h *ex vivo* perfusion. Upper row, photographs taken at 24-h. Leak of perfusate is seen from skin and muscle punch sites. Lower row, snapshots taken from videos recorded with near infrared camera in the first 30 s after injection. Limbs 1, 3 and 4 showed a homogenous distribution of ICG signal at the hooves Limb 2 and 5 showed less signal at the hoof. Heel and tibia showed homogenous distribution in all limbs, patchy areas of high intensity are from the ongoing leak from biopsy sites.

After ICG injection at 24 h, Limb 2 and Limb 5 showed less signal than the other limbs at the hoof level. Limbs 1, 3, and 4 exhibited homogeneous ICG signal distribution at the hoof level, indicating adequate distal perfusion. In contrast, Limbs 2 and 5 showed reduced signal intensity at the hooves. Across all limbs, the heel and tibial regions displayed uniform signal distribution, while localized areas of high intensity were attributed to ICG leakage from biopsy sites (Figure 11). In all limbs, a contraction response was seen after stimulation, while SCS limbs did not contract. Supplementary Video S1 demonstrates stimulation of Limb 5, before pedicle division (0–10 s), and at the end of the perfusion (10–23 s), and an SCS limb showing no stimulation response after temperature was increased to 25 °C (23–30 s).

## 4 Discussion

In this study, we established a 24-h *ex vivo* perfusion protocol for whole swine hindlimbs under sub-normothermic conditions (28 °C–32 °C) using a plasma-mimicking perfusate enriched with albumin and red blood cells. This strategy aimed to balance metabolic suppression with physiologically relevant oxygen delivery, thereby maintaining tissue viability without the heightened metabolic stress of full normothermia. This approach resulted in stable limb weight and peripheral resistance, preserved muscle morphology, conserved intracellular energy stores, sustained contractile responsiveness, and overall biochemical stability at the end of the perfusion period.

In our study, perfused limbs demonstrated minimal weight change, with an average of −0.53 ± 5.6% over 24 h of *ex vivo* perfusion. Weight gain during *ex vivo* perfusion is indicative of tissue injury and endothelial dysfunction. It has been shown that in swine forelimbs, a 5% weight gain is associated with significantly elevated myocyte injury scores, potassium, and lactate levels, while a 20% increase correlates with diminished contractility and compartment syndrome (24). Substantial weight gain has been reported in swine hindlimbs perfused under hypothermic conditions with low-potassium dextran solution, showing a 44% weight increase within 12 h at 30 mmHg pressure (33). The same group subsequently reported a 10% increase over 12 h with the same solution (18). Similarly, Steen solution (a dextran/albumin solution, with 7 g/dl albumin and 5 g/L Dextran-40 as colloids developed for *ex vivo* lung perfusion) use in swine forelimbs resulted in a 41% increase at 30 mmHg pressure over 24 h (34) while trans-humeral human extremities perfused with Steen Solution at a similar pressure resulted in 4.3% increase. lower sub-normothermic (20 °C–25 °C) settings, partial swine hindlimbs, perfused at 21 °C and 40 mmHg with a modified Steen solution (augmented with additional bovine serum albumin at 15 g/dl instead of 7 g/dl human albumin, overall increasing the colloidal pressure) still gained 14.5% in weight (35). In another study, the same solution was used in the same model to study pulsatile and continuous flows, resulting in 12.43% and 14.48% increases, respectively (36). In the temperature range similar to ours (27 °C–32 °C), oxygen carriers have been used either as whole blood or red blood cell concentrates. Constantinescu et al. (37) perfused swine forelimbs at 32 °C with 100–150 ml/min flow (Corresponding to 33 mmHg) using a hydroxyethyl starch and whole blood mixture and reported a 1.32% weight increase over 12 h. Ozer et al. (25) perfused swine forearms at 27 °C–32 °C with washed red blood cells, plasma and dextran solution and reported 20% weight increase over 24-h. Werner et al. (38) perfused human forearms with a plasma based perfusate with red blood cells at 30 °C–33 °C at 93 ± 2 mmHg pressure (not exceeding 110 mmHg) and observed −0.4% weight loss on average. Under normothermic conditions, red blood cells and HBOC-201 have been used with normotensive pressures. Fahradyan et al. (29) perfused swine total forelimbs using red blood cells, Plasmalyte-A and colloid solution with a physiologic concentration of ablumin at physiological perfusion pressure. Perfusion was done until systolic arterial pressure 115 mmHg and above, fullness of compartments or drop of tissue oxygen saturation by 20%. These failure signs were met at a median of 25 h (range 24–44 h) with an average 7.28% weight increase and final average compartment pressure of 24 mmHg. In a subsequent study, HBOC-201 was compared to RBC-based perfusion under the same endpoint settings. HBOC-201 group lasted 22.5 ± 1.7 h, while the RBC group lasted 28.2 ± 7.3 h, with 23.1% and 13.18% weight increases, respectively (39). The same approach was also used in the human trans-humeral model, with perfusion lasting 41.6 ± 9.4 h, with 0.4 ± 12.2% weight change, and 21.7 ± 15.5 mmHg flexor compartment pressure (19).

Our perfusate base (LPD + 2.5 g/dl albumin) has been formulated to have around twice the oncotic pressure of plasma and has a lower albumin concentration than the reported other custom formulations for VCA perfusion like Steen + that has been used at 21 °C without oxygen carriers with around 15% weight increase over 24-h (35, 36, 40, 41). Higher oncotic pressure, combined with low-flow conditions, controlled edema over extended perfusion durations in our studies. A recent meta-analysis shows that weight increase is not correlated with albumin concentration, and perfusions done with red blood cells as oxygen carriers show significantly less weight increase than acellular compositions and flow per unit tissue is correlated with weight increase (42).

Our model involves intact leg fascial compartments, allowing for the simulation of clinically relevant limb perfusions, enabling the study of compartmental perfusion dynamics. In swine, total forelimb model also involves intact compartments as studied in normothermic-normotensive perfusions with increases of the compartment pressures (24, 29, 39, 43–45). Partial hindlimb model involving the knee (35, 36) or forelimbs that are amputated at the elbow (25) does include confined fascial spaces and weight increases in these models might not have an effect on resistance or can obscure tissue damage to some extent. Recently, Oubari et al. established a non-human primate model that has intact compartments, they noted in their studies that around 10% weight increase the limbs deteriorates, they were then able to keep weight increase at 8% over 24-h with a combination of high oncotic Steen + solution and low stable flow parameters (46). In our model repeated sampling (6 mm punch for proximal, 3 mm punch for distal sites, for every 6 h) over two biopsy sites may have confounded the weight measurements. Moreover, the open musculature of the thigh also limits any resistance build-up to the muscle. The rise in compartment pressure observed in our experiments (from 8.4 ± 3.4 mmHg at baseline to 16.6 ± 2.9 mmHg at 24 h) is likely attributable to two factors. First, repeated pressure measurements from the same site and intermittent system flushes may have incrementally increased local pressure in the absence of physiological clearance mechanisms. Second, the *ex vivo* environment lacks neurohumoral regulation of vascular tone, which may permit progressive microvascular congestion and some pressure elevation despite stable limb weight. Notably, increased compartment pressure in the absence of weight gain has also been reported in normothermic perfusion studies. In porcine forelimbs perfused for 12 h, weight decreased by 1.2%, yet anterior and posterior compartment pressures averaged 16.5 ± 8.6 mmHg. This observation should be further studied in compartment intact models across different strategies. However, the values remained below clinically relevant thresholds. This increase may have been influenced by repeated measurements in the same region, potentially causing local trauma or fluid leakage.

Excessive edema during prolonged perfusion periods (>12 h) may compromise tissue quality and significantly reduce the feasibility of orthotopic transplantation. Our findings suggest that the oncotic support provided by our perfusate base and the oxygen-carrying capacity of red blood cells played a critical role in maintaining endothelial function and tissue integrity.

In addition to the absence of edema, our findings indicate that *ex vivo* perfusion supports sustained energy metabolism in extremity skeletal muscle, as evidenced by preserved ATP levels over time at both biopsy sites. Similar observation are seen in VCA and other organ perfusions. Pendexter et al. (47) showed an increase of ATP after subnormothermic machine perfusion in the rat limb model, along with preserved energy charge, reflecting a ratio of high-energy phosphates similar to healthy controls. Similar increases in ATP following machine perfusion have also been reported in liver (48) and kidney (49). ATP is increased after machine perfusions. In contrast, static cold storage showed a progressive decline in ATP levels, emphasizing that cold ischemia at 4 °C cannot fully arrest metabolic activity. Although early differences between groups at 6 h were not statistically significant, by 12 h, perfused limbs exhibited substantially higher ATP levels, particularly in distal muscle samples. This trend suggests a meaningful biological advantage of continuous perfusion, especially in preparation for reperfusion. Notably, ATP levels in perfused tissues remained more than twice those observed in static cold storage controls, underscoring the potential of perfusion-based strategies to mitigate ischemic energy depletion and extend the metabolic viability of preserved limbs.

Corroborating the absence of edema and preservation of energy stores, morphologic assessment of muscle in perfused limbs revealed no significant deterioration compared to baseline. Under ischemic conditions, ATP depletion leads to failure of the Na^+^/K^+^ ATPase pump, causing intracellular sodium accumulation and osmotic myocyte swelling. This disrupts cellular architecture and contributes to early structural damage (50). However, both proximal and distal biopsy sites in perfused limbs showed no significant increase in injury scores over time, indicating consistent and effective perfusion throughout the limb. In contrast, static cold storage demonstrated structural alterations by 12 h, highlighting the limitations of traditional preservation approaches. Although early differences between groups were not statistically significant, the sustained preservation of muscle architecture supports the potential of our protocol to reduce ischemic injury and extend the viable preservation window for composite tissues. We further support these histomorphological findings by demonstrating a well-preserved microvascular architecture, as evidenced by a conserved CD31-positive capillary network in muscle sections after 24 h of *ex vivo* perfusion. CD31 immunoreactivity remained comparable to baseline, indicating maintenance of endothelial integrity and vascular continuity throughout the perfusion period. The persistence of intact capillary structures suggests that our perfusion strategy was sufficient to prevent endothelial denudation or ischemia-related capillary loss, all of which are hallmarks of early tissue injury. This preservation of the capillary bed aligns with the stable ATP levels, minimal edema, and favorable metabolic parameters observed, collectively supporting sustained tissue viability over the 24-h perfusion interval. We were able to demonstrate muscle contractions in our study. While muscle contraction is influenced by temperature (51), electrolyte balance (52), and pH (53), and therefore cannot serve as a direct measure of overall limb viability (24), it remains a valuable functional marker. The ability to elicit contraction reflects preserved nerve conduction, as well as intact muscle depolarization and repolarization—processes that depend on a permissive window of pH, potassium, sodium, and calcium (54). Although contraction strength was not graded due to the inherent subjectivity and variability introduced by limb positioning, the presence of a contractile response provides qualitative evidence of preserved neuromuscular function during extended *ex vivo* perfusion.

It is important to highlight the changes observed in lactate, sodium, calcium, and free hemoglobin levels in our perfusate samples over time, as these parameters offer insight into tissue metabolism, cellular stress, and red blood cell integrity during prolonged *ex vivo* perfusion. Venous concentrations of lactate, sodium, and calcium increased significantly throughout the perfusion period, which likely reflects a combination of ongoing cellular stress and the absence of physiologic clearance mechanisms in the closed-loop system. The rise in sodium can be attributed to pH corrections with sodium bicarbonate, which likely distorted the readouts. Alternative buffers like tromethamine can be used to avoid sodium increase. *In vivo*, these metabolites would be continuously cleared by the liver and kidneys, which are not present in the *ex vivo* perfusion setup (55). This emphasizes the potential value of more robust perfusate exchanges or adjunctive strategies to reduce tissue injury and metabolite buildup. However, the normalized production of lactate, K^+^ and Ca^+2^, surrogates for tissue injury, were initially higher due to the preceding ischemic period (ongoing release from the limb) but declined and remained stable for most of the perfusion, suggesting adequate oxygenation and tissue perfusion. Oxygen consumption was calculated to be highest at baseline, although this difference was not statistically significant. This early elevation was mainly due to the initial perfusate pass through previously ischemic tissue, as the first samples were taken immediately. As the system stabilized, oxygen consumption decreased and remained steady thereafter. In humans, muscle oxygen uptake is measured at 0.31–4.3 ml/min.kg at rest and up to 42 ml/min.kg with exercise using diffuse optical spectroscopy or MRI (56–58). Our calculations were 0.9 ml/min.kg at baseline and 0.4 ml/min.kg at the endpoint, within the sub-normothermic range, similar to the range of resting-state muscle. In other reported VCA perfusion studies under normothermia, uptake rates up to 62 ml/min.kg have been reported in the swine forelimb model. In that study, the conditions were normotensive and hourly muscle stimulations were performed (24). Human forearm oxygen uptake was reported to average 5.7 ± 2.8 ml/min.g under *ex vivo* normothermic perfusion (19). In this value, there is likely a unit conversion problem because it exceeds even a professional athlete's total body VO2max (Oxygen consumption under exercise), which is around 50–80 ml/min.kg, by a factor of 100 (59). Consistency in units, flow-resistance calculations, and calculation formulas will be essential for further study of oxygen consumption across strategies. Studies that focus on oxygen delivery during *ex vivo* perfusion using more sophisticated methods would provide a robust framework for understanding metabolic responses at different temperatures.

In our model, which used autologous red blood cells as oxygen carriers, free hemoglobin levels rose significantly after 12 h, reaching 6.56 ± 1.57 mg/dl and increasing to 11.88 ± 3.41 mg/dl by the end of the 24-h perfusion. In line pressure was maintained at 60–65 mmHg and vascular pressure between 40 and 50 mmHg using a roller pump. Although several studies have used similar perfusion conditions, most have not reported free hemoglobin levels, despite the likelihood of comparable or greater hemolysis (19, 24, 29, 31, 39, 43, 60). Among identified studies that used red blood cells for *ex vivo* limb perfusion, only one reported free hemoglobin accumulation, which exceeded 80 mg/dl at 24 h (38). It is important to note that free hemoglobin concentrations are influenced by total circulating volume, which varies across models. In our setup, the perfusate volume was limited due to the use of autologous blood, restricting our ability to perform volume exchanges that could have attenuated this rise. Alternative strategies, such as incorporating centrifugal pumps or filtration systems, may help reduce hemolysis and warrant further investigation in future protocols.

This study has several limitations that should be acknowledged. First, the absence of a reperfusion phase prevents assessment of how well the preserved tissue would tolerate revascularization and recovery. Additionally, viability evaluation was limited to muscle tissue; no dedicated assessments were performed for skin, nerve, or vascular endothelium, which are essential components of vascularized composite tissue grafts. While muscle contraction was demonstrated, it was not quantified due to variability in limb positioning, and electromyographic or electrophysiologic studies were not performed. Moreover, we could estimate organ resistance *post hoc* by calibrating our line pressure measurements. For future studies, calibration measurements will precede the perfusion for each experiment. Direct probing of the artery would be the gold standard; however, in most *ex vivo* perfusion circuits, measurements are taken from the line.

In future work, we aim to expand viability assessment to additional tissue types, including skin, nerve, and vasculature, to provide a more comprehensive evaluation of graft preservation. Electromyography performed during perfusion may help clarify the impact of *ex vivo* preservation on nerve conduction. As nerve regeneration is critical for functional recovery following limb transplantation, comparing regenerative potential between *ex vivo*–perfused and cold-stored limbs could provide valuable insights. Efforts will also focus on protocol optimization to enable larger volume exchanges—potentially using pooled donor blood or compatible alternatives—to better maintain perfusion conditions. In addition, the protocol will be advanced toward application in replantation or transplantation models.

Although *ex vivo* perfusion has demonstrated superiority over static cold storage across different temperature conditions in VCA models, the next step for the field should involve direct, head-to-head comparisons of perfusion strategies at predefined time points (e.g., 6, 12, 18, 24 h and beyond), rather than evaluating each approach in isolation. This should include systematic comparison of cellular (RBC-based) vs. acellular perfusates to determine whether time-dependent thresholds exist at which one strategy becomes superior in terms of edema control, metabolic stability, and tissue integrity.

## 5 Conclusion

Extended (24 h) sub-normothermic (28 °C–32 °C) *ex vivo* perfusion of a large extremity composite allograft using modified low potassium dextran solution with a low hematocrit (donor red blood cells) is feasible and demonstrates significant advantages over cold storage both metabolically and morphologically. The results mark progress toward developing clinically translatable perfusion protocols that could enhance the success and availability of vascularized composite allotransplantation.

## Data Availability

The raw data supporting the conclusions of this article will be made available by the authors, without undue reservation.

## References

[1] Fitzgibbons P Medvedev G. Functional and clinical outcomes of upper extremity amputation. J Am Acad Orthop Sur. (2015) 23:751–60. 10.5435/JAAOS-D-14-00302 26527583

[2] Wells MW Rampazzo A Papay F Gharb BB. Two decades of hand transplantation: a systematic review of outcomes. Ann Plast Surg. (2022) 88:335–44. 10.1097/SAP.0000000000003056 35113506

[3] Bernardon L Gazarian A Petruzzo P Packham T Guillot M Guigal V Bilateral hand transplantation: functional benefits assessment in five patients with a mean follow-up of 7.6 years (range 4–13 years). J Plast Reconstr Aesthet Surg. (2015) 68:1171–83. 10.1016/j.bjps.2015.07.007 26297387

[4] Jing L Yao L Zhao M Peng L-P Liu M. Organ preservation: from the past to the future. Acta Pharmacol Sin. (2018) 39(5):845–57. 10.1038/aps.2017.182 29565040 PMC5943901

[5] Paradis S Charles AL Meyer A Lejay A Scholey JW Chakfé N Chronology of mitochondrial and cellular events during skeletal muscle ischemia-reperfusion. Am J Physiol Cell Physiol. (2016) 310(11):C968–82. 10.1152/ajpcell.00356.2015 27076618 PMC4935201

[6] Slegtenhorst BR Dor FJ Rodriguez H Voskuil FJ Tullius SG. Ischemia/reperfusion injury and its consequences on immunity and inflammation. Curr Transplant Rep. (2014) 1(3):147–54. 10.1007/s40472-014-0017-6 25419507 PMC4235532

[7] Thomason PR Matzke HA. Effects of ischemia on the hind limb of the rat. Am J Phys Med. (1975) 54(3):113–31. 1137010

[8] He J Khan UZ Qing L Wu P Tang J. Improving the ischemia-reperfusion injury in vascularized composite allotransplantation: clinical experience and experimental implications. Front Immunol. (2022) 13:998952. 10.3389/fimmu.2022.998952 36189311 PMC9523406

[9] Chakradhar A Mroueh J Talbot SG. Ischemia time in extremity allotransplantation: a comprehensive review. Hand (N Y). (2024) 21(1):12–20. 10.1177/15589447241287806 39558824 PMC11574782

[10] Stevanovic M Sharpe F. Functional free muscle transfer for upper extremity reconstruction. Plast Reconstr Surg. (2014) 134(2):257e–74e. 10.1097/PRS.0000000000000405 24732655

[11] Perera T Mergental H Stephenson B Roll GR Cilliers H Liang R First human liver transplantation using a marginal allograft resuscitated by normothermic machine perfusion. Liver Transpl. (2016) 22:120–4. 10.1002/lt.24369 26566737

[12] Nicholson ML Hosgood SA. Renal transplantation after *ex vivo* normothermic perfusion: the first clinical study. Am J Transplant. (2013) 13(5):1246–52. 10.1111/ajt.12179 23433047

[13] Villanueva JE Joshi Y Emmanuel S Gao L Macdonald PS. Expanding donor heart utilization through machine perfusion technologies. Curr Transplant Rep. (2022) 9:219–26. 10.1007/s40472-022-00375-0

[14] Cypel M Yeung JC Hirayama S Rubacha M Fischer S Anraku M Technique for prolonged normothermic *ex vivo* lung perfusion. J Heart Lung Transplant. (2008) 27(12):1319–25. 10.1016/j.healun.2008.09.003 19059112

[15] Cypel M Yeung JC Liu M Anraku M Chen F Karolak W Normothermic *ex vivo* lung perfusion in clinical lung transplantation. N Engl J Med. (2011) 364(15):1431–40. 10.1056/NEJMoa1014597 21488765

[16] Machuca TN Mercier O Collaud S Tikkanen J Krueger T Yeung JC Lung transplantation with donation after circulatory determination of death donors and the impact of *ex vivo* lung perfusion. Am J Transplant. (2015) 15(4):993–1002. 10.1111/ajt.13124 25772069

[17] Mergental H Perera MTPR Laing RW Muiesan P Isaac JR Smith A Transplantation of declined liver allografts following normothermic ex-situ evaluation. Am J Transplant. (2016) 16(11):3235–45. 10.1111/ajt.13875 27192971

[18] Kueckelhaus M Dermietzel A Alhefzi M Aycart MA Fischer S Krezdorn N Acellular hypothermic extracorporeal perfusion extends allowable ischemia time in a porcine whole limb replantation model. Plast Reconstr Surg. (2017) 139(4):922e–32e. 10.1097/PRS.0000000000003208 28350667

[19] Rezaei M Ordenana C Figueroa BA Said SA Fahradyan V Dalla Pozza E *Ex vivo* normothermic perfusion of human upper limbs. Transplantation. (2022) 106(8):1638–46. 10.1097/TP.0000000000004045 35152257

[20] Gok E Alghanem F Moon R Guy E Rojas-Pena A Bartlett RH Development of an ex-situ limb perfusion system for a rodent model. ASAIO J. (2019) 65(2):167–72. 10.1097/MAT.0000000000000786 29595532 PMC6158126

[21] Ozer K Rojas-pena A Mendias CL Bryner BS Toomasian C Bartlett RH. The effect of ex situ perfusion in a swine limb. J Hand Surg. (2016) 41(1):3–12. 10.1016/j.jhsa.2015.11.003 26710728

[22] Burlage LC Lellouch AG Taveau CB Tratnig-frankl P Pendexter CA Randolph MA Optimization of *ex vivo* machine perfusion and transplantation of vascularized composite allografts. J Surg Res. (2023) 270:151–61. 10.1016/j.jss.2021.09.005 PMC8712379 34670191

[23] Duru Ç Biniazan F Hadzimustafic N D’Elia A Shamoun V Haykal S. Review of machine perfusion studies in vascularized composite allotransplant preservation. Front Transplant. (2023) 2:1323387. 10.3389/frtra.2023.1323387 38993931 PMC11235328

[24] Meyers A Pandey S Kopparthy V Sadeghi P Clark RC Figueroa B Weight gain is an early indicator of injury in *ex vivo* normothermic limb perfusion (EVNLP). Artif Organs. (2023) 47(2):290–301. 10.1111/aor.14442 36305734 PMC10100395

[25] Ozer K Rojas-Pena A Mendias CL Bryner B Toomasian C Bartlett RH. Ex situ limb perfusion system to extend vascularized composite tissue allograft survival in swine. Transplantation. (2015) 99(10):2095–101. 10.1097/TP.0000000000000756 25929606

[26] Kruit AS Brouwers K van Midden D Zegers H Koers E van Alfen N Successful 18-h acellular extracorporeal perfusion and replantation of porcine limbs—histology versus nerve stimulation. Transpl Int. (2021) 34(2):365–75. 10.1111/tri.13802 33316847 PMC7898521

[27] Landin L Cavadas PC Garcia-Cosmes P Thione A Vera-Sempere F. Perioperative ischemic injury and fibrotic degeneration of muscle in a forearm allograft: functional follow-up at 32 months post transplantation. Ann Plast Surg. (2011) 66(2):202–9. 10.1097/SAP.0b013e318206a365 21200306

[28] Yamazaki F Yokomise H Keshavjee SH Miyoshi S Cardoso PF Slutsky AS He superiority of an extracellular fluid solution over euro-collins’ solution for pulmonary preservation. Transplantation. (1990) 49(4):690–4. 10.1097/00007890-199004000-00007 1691536

[29] Fahradyan V Said SAD Ordenana C Dalla Pozza E Frautschi R Duraes EFR Extended *ex vivo* normothermic perfusion for preservation of vascularized composite allografts. Artif Organs. (2020) 44(8):846–55. 10.1111/aor.13678 32133657

[30] Boody AR Wongworawat MD. Accuracy in the measurement of compartment pressures: a comparison of three commonly used devices. J Bone Joint Surg Am. (2005) 87(11):2415–22. 10.2106/JBJS.D.02826 16264116

[31] Duraes EFR Madajka M Frautschi R Soliman B Cakmakoglu C Barnett A Developing a protocol for normothermic ex-situ limb perfusion. Microsurgery. (2018) 38(2):185–94. 10.1002/micr.30252 28990205

[32] Brouwers K van Geel SRWM van Midden D Kruit AS Kusters B Hummelink S Added value of histological evaluation of muscle biopsies in porcine vascularized composite allografts. J Clin Med. (2024) 13(17):5167. 10.3390/jcm13175167 39274379 PMC11395792

[33] Kueckelhaus M Fischer S Sisk G Kiwanuka H Bueno EM Dermietzel A A mobile extracorporeal extremity salvage system for replantation and transplantation. Ann Plast Surg. (2016) 76(3):355–60. 10.1097/SAP.0000000000000681 26808757

[34] Krezdorn N Macleod F Tasigiorgos S Turk MDM Wo L Kiwanuka BAH Twenty-four-hour *ex vivo* perfusion with acellular solution enables successful replantation of porcine forelimbs. Plast Reconstr Surg. (2019) 144(4):608e–18e. 10.1097/PRS.0000000000006084 31568296

[35] Goutard M Tawa P Berkane Y Andrews AR Pendexter CA de Vries RJ Machine perfusion enables 24-h preservation of vascularized composite allografts in a swine model of allotransplantation. Transpl Int. (2024) 37:12338. 10.3389/ti.2024.12338 38813393 PMC11133529

[36] Tawa P Goutard M Andrews AR de Vries RJ Rosales IA Yeh H Continuous versus pulsatile flow in 24-h vascularized composite allograft machine perfusion in swine: a pilot study. J Surg Res. (2023) 283:1145–53. 10.1016/j.jss.2022.11.003 36915006 PMC10867902

[37] Constantinescu MA Knall E Xu X Kiermeir DM Jenni H Gygax E Preservation of amputated extremities by extracorporeal blood perfusion; a feasibility study in a porcine model. J Surg Res. (2011) 171(1):291–9. 10.1016/j.jss.2010.01.040 20451920

[38] Werner NL Alghanem F Rakestraw SL Sarver DC Nicely B Pietroski RE Ex situ perfusion of human limb allografts for 24 hours. Transplantation. (2017) 101(3):e68–74. 10.1097/TP.0000000000001500 28222055

[39] Figueroa BA Said SA Ordenana C Rezaei M Orfahli LM Dubé GP *Ex vivo* normothermic preservation of amputated limbs with a hemoglobin-based oxygen carrier perfusate. J Trauma Acute Care Surg. (2022) 92(2):388–97. 10.1097/TA.0000000000003395 34510075

[40] Goutard M De Vries RJ Tawa P Pendexter CA Rosales IA Tessier SN Exceeding the limits of static cold storage in limb transplantation using subnormothermic machine perfusion. J Reconstr Microsurg. (2023) 39(5):350–60. 10.1055/a-1886-5697 35764315 PMC10848168

[41] Charlès L Filz Von Reiterdank I Lancia HH Shamlou AA Berkane Y Rosales I Effect of subnormothermic machine perfusion on the preservation of vascularized composite allografts after prolonged warm ischemia. Transplantation. (2024) 108(11):2222–32. 10.1097/TP.0000000000005035 38722685 PMC11518650

[42] Marlar R Abbas F Obeid R Frisbie S Ghazoul A Rezaee A A meta-analysis of perfusion parameters affecting weight gain in *ex vivo* perfusion. Artif Organs. (2024) 49(1):7–20. 10.1111/aor.14841 39157933 PMC11687208

[43] Rohde E Goudarzi M Madajka M Said SAD Ordenana C Rezaei M Metabolic profiling of skeletal muscle during ex-vivo normothermic limb perfusion. Mil Med. (2021) 186(Suppl 1):358–63. 10.1093/milmed/usaa268 33499445

[44] Said SA Ordeñana CX Rezaei M Figueroa BA Dasarathy S Brunengraber H Ex-vivo normothermic limb perfusion with a hemoglobin-based oxygen carrier perfusate. Mil Med. (2020) 185(Suppl 1):110–20. 10.1093/milmed/usz314 32074378 PMC8204701

[45] Knoedler L Klimitz FJ Huelsboemer L Niederegger T Schaschinger T Knoedler S Experimental swine models for vascularized composite allotransplantation and immunosuppression: a systematic review and case report of a novel heterotopic hemifacial swine model. Transpl Int. (2025) 38:14520. 10.3389/ti.2025.14520 40799314 PMC12341719

[46] Oubari H Van Dieren L Berkane Y Cabanel L Randolph MA Cetrulo C Development of a 24-h preservation protocol of forearm vascularized composite allotransplants in nonhuman primates using subnormothermic machine perfusion. Transplant Direct. (2025) 11(9):e1849. 10.1097/TXD.0000000000001849 40785850 PMC12333806

[47] Pendexter CA Haque O Mojoudi M Maggipinto S Goutard M Baicu S Development of a rat forelimb vascularized composite allograft (VCA) perfusion protocol. PLoS One. (2023) 18(1):e0266207. 10.1371/journal.pone.0266207 36652460 PMC9847903

[48] Bruinsma BG Sridharan GV Weeder PD Avruch JH Saeidi N Özer S Metabolic profiling during *ex vivo* machine perfusion of the human liver. Sci Rep. (2016) 6:22415. 10.1038/srep22415 26935866 PMC4776101

[49] Kawamura M Parmentier C Ray S Clotet-Freixas S Leung S John R Normothermic *ex vivo* kidney perfusion preserves mitochondrial and graft function after warm ischemia and is further enhanced by AP39. Nat Commun. (2024) 15(1):8086. 10.1038/s41467-024-52140-9 39278958 PMC11402965

[50] Blaisdell FW. The pathophysiology of skeletal muscle ischemia and the reperfusion syndrome: a review. Cardiovascular Surgery. (2002) 10(6):620–30. 10.1016/s0967-2109(02)00070-4 12453699

[51] Ranatunga KW. Temperature effects on force and actin-myosin interaction in muscle: a look back on some experimental findings. Int J Mol Sci. (2018) 19:1538. 10.3390/ijms19051538 29786656 PMC5983754

[52] McKenna MJ Renaud JM Ørtenblad N Overgaard K. A century of exercise physiology: effects of muscle contraction and exercise on skeletal muscle Na+,K+-ATPase, Na+ and K+ ions, and on plasma K+ concentration—historical developments. Eur J Appl Physiol. (2024) 124:681–751. 10.1007/s00421-023-05335-9 38206444 PMC10879387

[53] Debold EP Beck SE Warshaw DM. Effect of low pH on single skeletal muscle myosin mechanics and kinetics. Am J Physiol Cell Physiol. (2008) 295:173–9. 10.1152/ajpcell.00172.2008 PMC2493560 18480297

[54] Calderón JC Bolaños P Caputo C. The excitation–contraction coupling mechanism in skeletal muscle. Biophys Rev. (2014) 6(1):133–60. 10.1007/s12551-013-0135-x 28509964 PMC5425715

[55] Huang T Liang Z Wang K Miao X Zheng L. Novel insights into athlete physical recovery concerning lactate metabolism, lactate clearance and fatigue monitoring: a comprehensive review. Front Physiol. (2025) 16:1459717. 10.3389/fphys.2025.1459717 40200988 PMC11975961

[56] Boebinger SE Brothers RO Bong S Sanders B McCracken C Ting LH Diffuse optical spectroscopy assessment of resting oxygen metabolism in the leg musculature. Metabolites. (2021) 11(8):496. 10.3390/metabo11080496 34436437 PMC8400025

[57] Henry B Zhao M Shang Y Uhl T Thomas DT Xenos ES Hybrid diffuse optical techniques for continuous hemodynamic measurement in gastrocnemius during plantar flexion exercise. J Biomed Opt. (2015) 20(12):125006. 10.1117/1.JBO.20.12.125006 26720871 PMC4688865

[58] Zheng J An H Coggan AR Zhang X Bashir A Muccigrosso D Noncontrast skeletal muscle oximetry. Magn Reson Med. (2014) 71(1):318–25. 10.1002/mrm.24669 23424006 PMC3661680

[59] Srivastava S Tamrakar S Nallathambi N Vrindavanam SA Prasad R Kothari R. Assessment of maximal oxygen uptake (VO2 Max) in athletes and nonathletes assessed in sports physiology laboratory. Cureus. (2024) 16(5):e61124. 10.7759/cureus.61124 38919211 PMC11197041

[60] Amin KR Stone JP Kerr J Geraghty A Joseph L Montero-Fernandez A Randomized preclinical study of machine perfusion in vascularized composite allografts. Br J Surg. (2021) 108(5):574–82. 10.1002/bjs.11921 34043778 PMC10364870

