# Supplementary material for: 24-hour ex vivo perfusion of vascularized composite allografts in a large animal total limb model
Source: Front Transplant. 2026 Feb 12;5:1718484. doi: 10.3389/frtra.2026.1718484 (PMC12935989; doi:10.3389/frtra.2026.1718484)
Supplement: Supplementary file 1 [file Table1.docx]

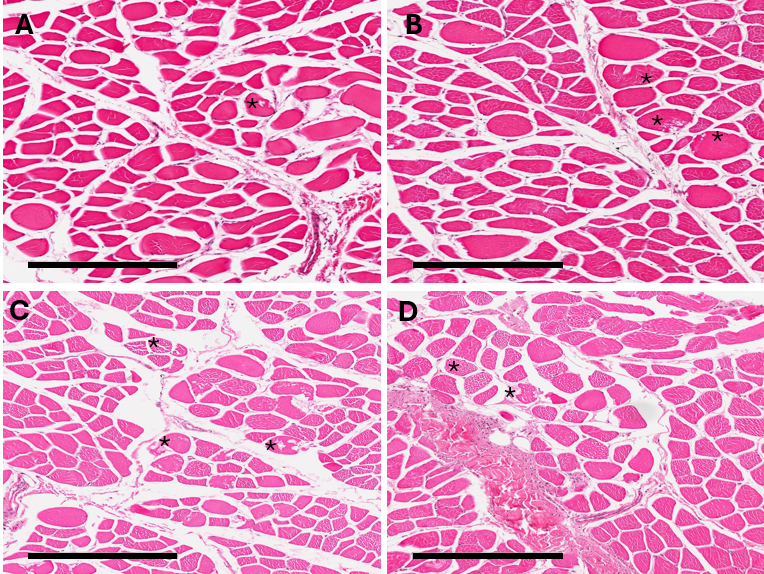


Figure 1. Proximal and Distal Muscle Samples after 24-hour Static Cold Storage with LPD

Severe variation and interfibrillar distance is seen as well ischemic fibers at various stages are seen in both Proximal (A&B) and Distal (C&D) samples Asterisk: Fragmented fibers. 10x H&E staining, Scale Bar: 300μm

**Table 1. Composition of the Low Potassium Dextran solution**

| Dextran 40 | 50g/L |
| --- | --- |
| Glucose | 5 mmol/L |
| Na^+^ | 138 mmol/L |
| K^+^ | 6 mmol/L |
| Mg^+2^ | 0.8 mmol/L |
| Chloride | 142 mmol/L |
| Sulfate | 0.8 mmol/L |
| Phosphates | 0.8 mmol/L |

25ml 1mmol/L Thromethamine (THAM) is provided with the solution and added to correct pH to 7.4

| Score | Edema | Variation | Damaged Fibers* |
| --- | --- | --- | --- |
| 0 | All muscle fibers stacked without any apparent space between fibers | All muscle fibers have the same shape and size | 0-5 myocytes |
| 1 | Minimal increase of interstitial space | Mild variation in diameter | 6-20 myocytes |
| 2 | Moderate increase of interstitial space | Moderate variation fiber diameter | 21-50 |
| 3 | Severe increase of interstitial space | Significant variation in fiber diameter | >51 |

**Table 2. Modified Histology Injury Severity Scores for Myocytes**

* Per 10 High Power Fields under 20x magnification. Damaged myoctes are defined as fibers with enlarged rounded fibers with hypereosinophilia, intracellular vacuoles, broken borders.

|  |  | Interstitial edema  Mean (SD) | Variation  Mean (SD) | Damaged myocytes  Mean (SD) | Total  Mean (SD) |
| --- | --- | --- | --- | --- | --- |
| Proximal muscles | | | | | |
| 0-hours | Perfusion | 1.53(0.49) | 1.23(0.48) | 0.26(0.34) | 3.03 (2.5-3.5) |
|  | Control | 1.48 (0.24) | 1.15 (0.32) | 0.2(0.21) | 2.4(0.56) |
| 6-hours | Perfusion | 1.83(0.11) | 1.06(0.19) | 0.4 (0.27) | 3.2 (0.7) |
|  | Control | 1.06(0.25) | 0.9 (0.19) | 0.2 (0.18) | 2.16(0.26) |
| 12-hours | Perfusion | 1.73(0.38) | 1.06 (0.40) | 0.2 (0.21) | 3 (0.8) |
|  | Control | 1.53 (0.29) | 1.3 (0.21) | 0.9(0.38) | 3.73 (0.75) |
| 18 hours | Perfusion | 1.5(0.62) | 1.06 (0.36) | 0.5 (0.23) | 3.06(1.1) |
| 24 hours | Perfusion | 1.76(0.57) | 1.23 (0.30) | 0.26(0.34) | 3.26(1.04) |
| Distal muscles | | | | | |
| 0-hours | Perfusion | 1.53 (0.75) | 1(0.57) | 0.36 (0.24) | 2.9 (1.34) |
|  | Control | 1.2(0.6) | 1 (0.37) | 0.53 (0.38) | 2.56 (0.69) |
| 6-hours | Perfusion | 2.1 (0.24) | 1.3 (0.23) | 0.7(0.5) | 4.2 (0.81) |
|  | Control | 1.6 (0.38) | 1.3 (0.18) | 0.63 (0.34) | 4(1.04) |
| 12-hours | Perfusion | 2.13 (0.27) | 1.5 (0.33) | 0.93 (0.56) | 4.56 (1.58) |
|  | Control | 1.7 (0.22) | 1.56 (0.27) | 0.9 (0.54) | 4 (1.04) |
| 18 hours | Perfusion | 1.88 (0.38) | 1.45 (0.51) | 1.1 (0.91) | 4.16 (1.41) |
| 24 hours | Perfusion | 1.86 (0.43) | 1.6 (0.38) | 1.67(0.56) | 4.63 (0.66) |

**Table 3. Histology injury severity scores of muscle samples**

**
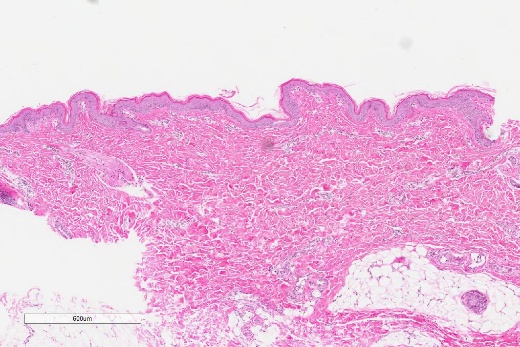

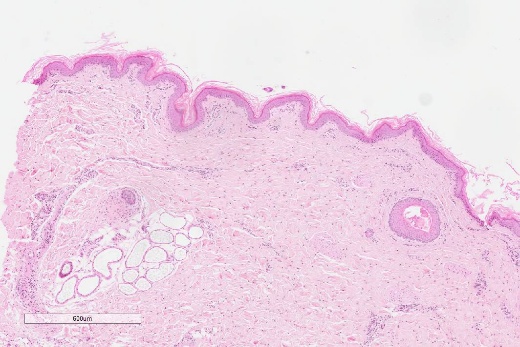

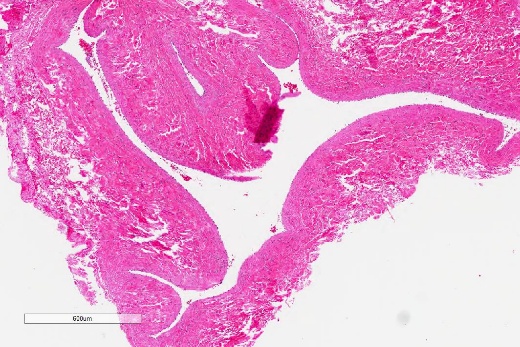

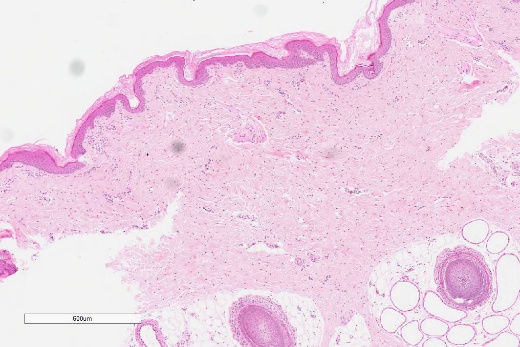

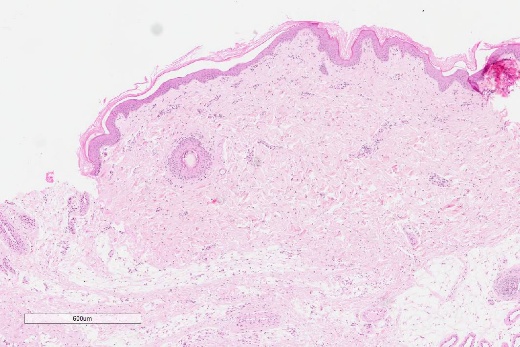

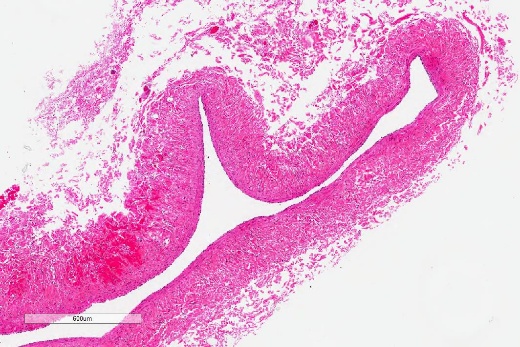

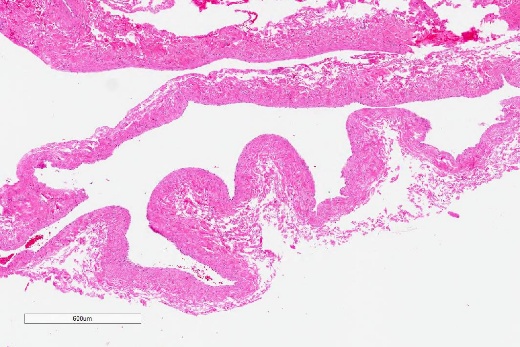

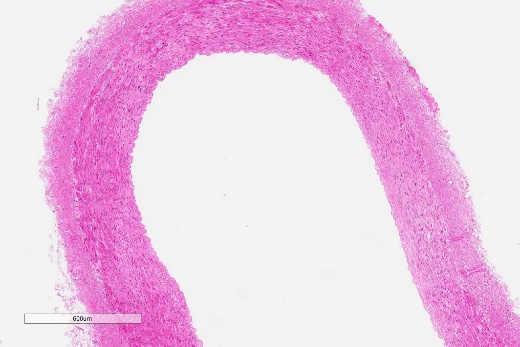

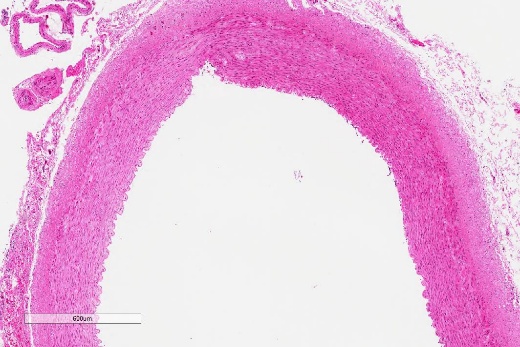

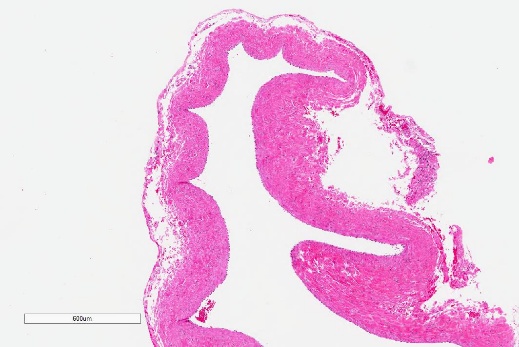

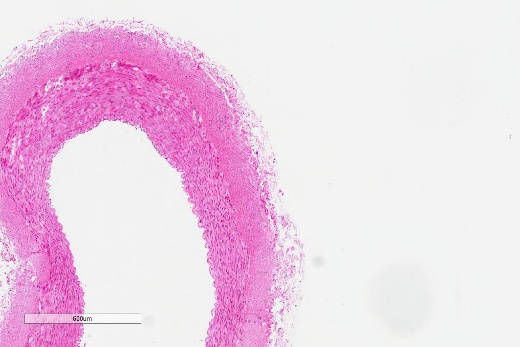

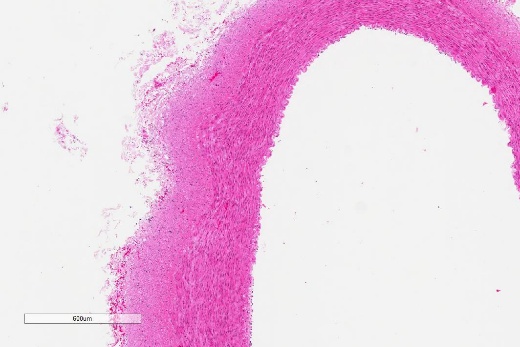

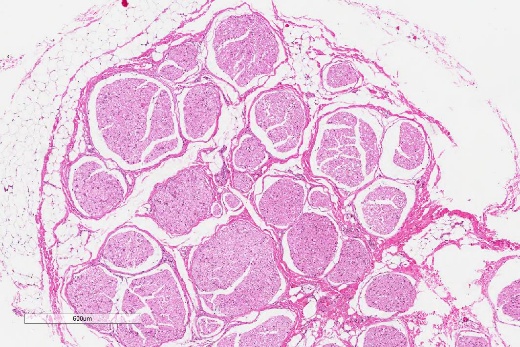

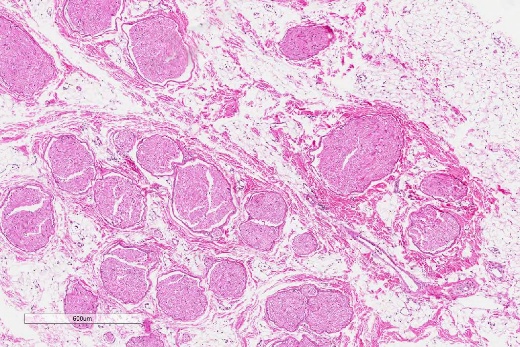

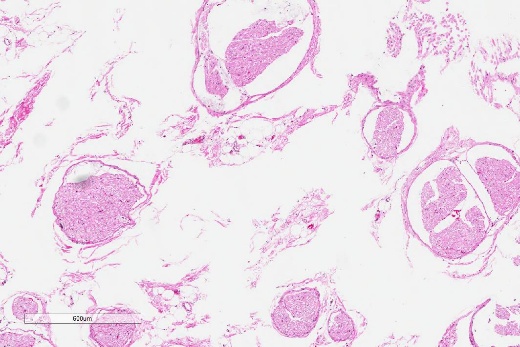

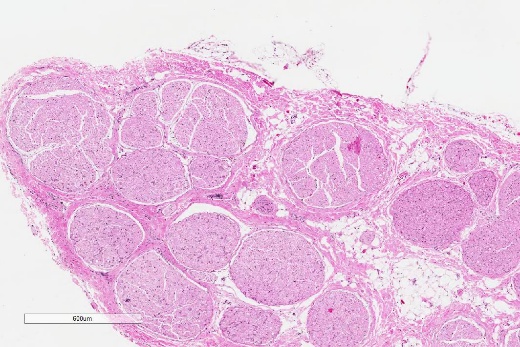
 Baseline Control Baseline Perfusion 12-h SCS 24-hour Perfusion**

*

**D**

**C**

**B**

**A**

*

*

*

*

*

*

*

*

*

*

*

*

*

**Figure 2 H&E staining, 4x in nerve, skin and vessels A)** Femoral Nerve, in 0-h baseline images in both groups epineurium and perineurium are regular, fascicles are in good contact with epineurium. In 12-SCS controls and 24-h perfusion groups interfascicular distances are increased (Arrow) and in some fascicles in cold storage group, the relationship between axon bundles and epineurium were lost (Asterisk). **B)** Femoral Artery, in 0-h baseline images intima (Arrow), media (Straight line) and adventitial layers (Asterisk) did not show any differences at 12-hour cold storage and 24-hour perfusion groups.**C)** Femoral vein, in 0-h baseline images intima (Arrow), media (Straight line) and adventitial layers (Asterisk) are seen and sections show folding due to less amount of muscle in veins in comparison to arteries. No clear differences were seen at 12-hour cold storage and 24-hour perfusion groups in comparison to baselines. **D)** Skin samples. At 0-h epidermis and dermis are seen in sections with hair follicles and glands (Asterisk). There were no structural differences at 12-hour cold storage and 24 hour perfusion groups. Scale Bar : 600μm


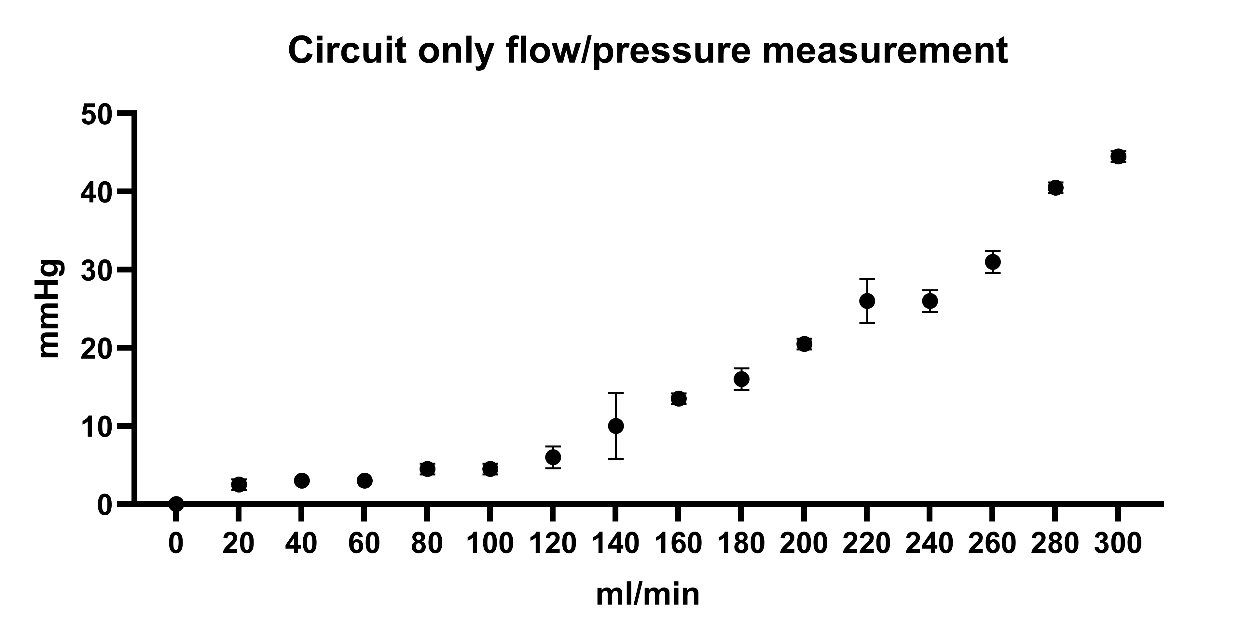


Figure3. Calibration curve for circuit only flow. Temperature set at 30°C, LPD with 2.5g/dL albumin used. Error bars represent SEM. Flow rates were fitted to the curve to estimate the decrease of pressure created by the endpoint cannula, then the values were subtracted to find estimated pressure to calculate the resistance.


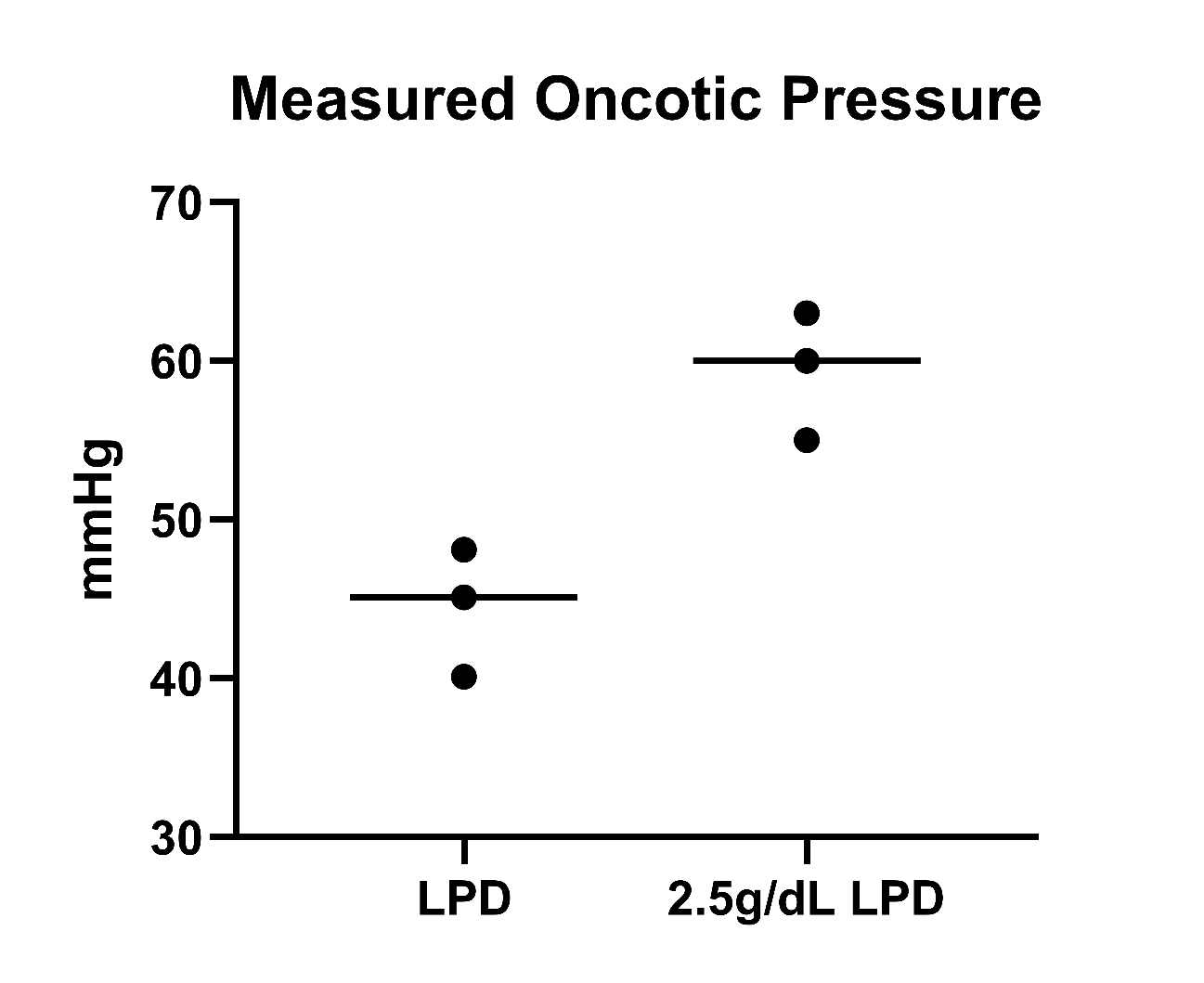


**Figure4**. **Oncotic Pressure Measurements.** Measurements were performed using a Oncometer device (*Onkometer BMT 923, Stahnsdorf Germany)*. Oncotic pressure of LPD was 44.43±4.04 and 59.33±4.0 mmHg for 2.5g/dL albumin enriched LPD. LPD= Low potassium dextran
